# Comparison of fruit quality and metabolome differences between three table grape varieties and their parents

**DOI:** 10.1016/j.fochx.2026.104567

**Published:** 2026-10-15

**Authors:** Liu Zhenghai, Xiao Feng, Wang Chenyu, Qiao Qinghe, Liu Jinpin, Tan Min, He Jinyu, Zhang Yanxia, Yang Xiaoyu, Lu Jiang, Ji Wei, Dong Zhigang, Zhao Qifeng

**Affiliations:** a College of Horticulture, Institute of Pomology, Shanxi Agricultural University, Taiyuan 030801, China; b School of Agriculture and Biology, Shanghai Jiao Tong University, Shanghai 200240, China

**Keywords:** Genetic analysis using SSR markers, Sensory evaluation, *Vitis*

## Abstract

To elucidate the differences in fruit quality and metabolome between table grape hybrid varieties and their parents, this study used ‘Kyoho’ (KG) as the female parent, ‘Zaoheibao’ (ZHB) as the male parent, and their three hybrid offspring ‘Baoguang’ (BG), ‘Cuiguang’ (CG), and ‘Miguang’ (MG) as materials. Basic fruit quality traits, total phenolic and anthocyanin contents were measured, sensory evaluation was conducted, and 30 pairs of SSR fluorescent primers were employed to verify the genetic relationships between parents and offspring. Meanwhile, LC-MS/MS-based untargeted metabolomics combined with multivariate statistical analysis was used to compare the metabolic profiles of the five varieties.The results showed that CG exhibited high soluble solids content (19.26%) and low titratable acidity (3.78 g·L^−1^), demonstrating excellent sugar-acid balance; BG exhibited the highest anthocyanin content (14.56 mg/100 g); MG possessed a strong rose aroma and achieved the highest overall sensory score. SSR molecular markers confirmed that all three offspring were true hybrids, and each variety was assigned a unique SSR barcode-based molecular identity. A total of 2038 metabolites were putatively annotated by metabolomics analysis. PCA and OPLS-DA confirmed clear metabolomic separation among varieties, with flavonoids and anthocyanins identified as key differential metabolites. The up- and down-regulation patterns of metabolites in the volcano plot indicate that the inheritance from the two parents in the hybrid progeny is not simply additive. The Euclidean distance between the parents KG and ZHB was the largest (77.70), whereas the distance between CG and KG was the smallest (62.17), indicating that CG had a metabolome more similar to that of the female parent KG. KEGG enrichment analysis indicated that differential metabolites were mainly enriched in amino acid biosynthesis and flavonoid metabolism pathways. Correlation analysis revealed that flavonol glycosides showed strong positive correlations with anthocyanins and total phenolics, while WGCNA analysis revealed that anthocyanins and total phenolics exhibited divergent dynamic trends. Redundancy analysis (RDA) showed that the first two axes cumulatively explained 74.39% of the covariance, revealing an opposing differentiation pattern between appearance quality and internal quality traits at the metabolic level. In conclusion, the three hybrid cultivars exhibit distinct genetic characteristics at both the fruit-quality and metabolomic levels, and amino-acid metabolic remodeling represents a plausible factor underlying their quality divergence.

## Highlights

• SSR barcodes confirmed three grape hybrids and their parental identities.
• Hybrid genetic effects are not simply additive.
• Amino-acid biosynthesis constitutes a key upstream pathway for quality differentiation.
• Amino-acid biosynthesis acts as a key upstream pathway for quality differentiation.

## **Graphical abstract**

Schematic overview of this study. Three table-grape hybrids (‘Baoguang’, BG; ‘Cuiguang’, CG; ‘Miguang’, MG) derived from the cross between female parent ‘Kyoho’ (KG) and male parent ‘Zaoheibao’ (ZHB) were investigated. SSR molecular markers validated their true-hybrid status and generated variety-specific DNA barcodes. Ripe berries were subjected to phenotypic characterization (fruit quality indices, total phenolic and anthocyanin quantification, sensory evaluation) and UHPLC-MS/MS-based untargeted metabolomics, yielding 2038 putatively annotated metabolites. Multivariate statistics, WGCNA, redundancy analysis and KEGG enrichment demonstrated that hybrid progenies exhibited **non-additive metabolic inheritance**, rather than a simple additive combination of parental metabolic traits. CG showed superior sugar-acid balance; BG accumulated the highest anthocyanin content; MG possessed prominent rose aroma and achieved the best sensory performance. Remodeling of amino-acid biosynthesis together with differential flavonoid and anthocyanin accumulation represents the critical metabolic basis for fruit-quality divergence among hybrids. Hybrid breeding can improve fruit quality and facilitate the selection of cultivars adapted to market demands.

**Figure:**
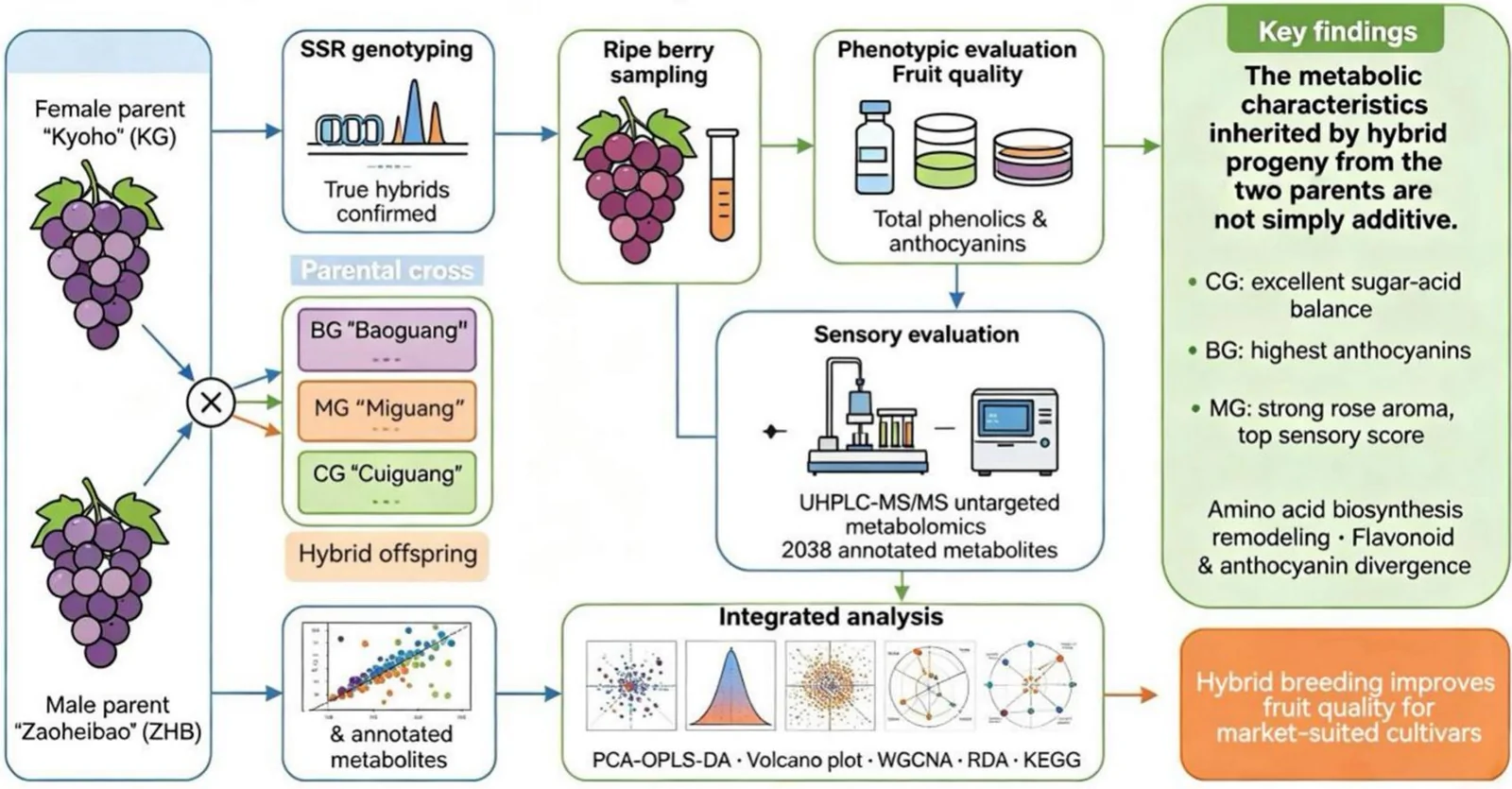

## 1 Introduction

Grape (*Vitis* spp.) is an economically important fruit crop widely cultivated worldwide. Its table grape varieties are favored by consumers due to their unique flavor and rich nutrition. With the improvement of living standards, the market demand for high-quality table grapes is increasing, requiring not only good external fruit quality (such as berry size, color, and bloom) but also greater attention to internal flavor and functional active components (Wang, Li, et al., 2025; Wang et al., 2023). Therefore, in-depth analysis of table grape fruit quality and its metabolite composition is of great significance for the breeding of superior varieties and quality regulation.

Cross breeding is an important approach for developing new grape varieties. Through genetic recombination of desirable traits from both parents, offspring with heterosis or specific characteristics can be generated (Guo et al., 2021; Yang et al., 2024). Hybrid offspring often exhibit complex segregation and recombination patterns in fruit quality traits (such as berry size, soluble solids, acidity, and aroma) and metabolite composition, rather than simple additive inheritance of parental traits (Wang, Yan, et al., 2025; Zhang, Zhang, et al., 2025). Metabolomics, as an important branch of systems biology, can comprehensively and qualitatively analyze the types and abundance changes of small-molecule metabolites in organisms (Li, Dou, et al., 2022; Lv et al., 2026), and has been widely applied in studies of fruit quality formation, variety identification, and metabolic pathway analysis in fruit trees (Li, Jiang, et al., 2022; Xue et al., 2025). Multi-omics analysis of overripe Cabernet Sauvignon berries revealed key enzyme genes and proteins associated with changes in metabolites such as glucose, pyruvate, malate, and alanine, providing a theoretical basis for optimizing grape harvest timing (Shi et al., 2025). Using the peel and pulp of the muscat-flavored ‘Shine Muscat’ and the neutral-flavored ‘Thompson Seedless’ at three developmental stages (young fruit, fruit softening, and maturity), correlation analysis of 46 differential compounds was performed, laying a theoretical foundation for understanding flavor formation in aromatic grapes (Li et al., 2025). Comparative omics analysis of four grape varieties revealed significantly higher levels of flavonoids in ‘Summer Black’, and both metabolomics and transcriptomics showed significant enrichment and up-regulation of the anthocyanin biosynthesis pathway (Zhang, Zhao, & Li, 2025). Using the Euro-American hybrid grape ‘Kyoho’ (KG) and other varieties, seed metabolites were comprehensively putatively annotated, inter-variety differences were compared, and the relationship between grape seed metabolites, skin color, and variety origin was explored (Sheng et al., 2023). Using an LC-HRMS based untargeted metabolomics approach, the metabolite composition of the Indian table grape hybrid ‘Manjari Medika’ (MM) and its parents ‘Pusa Navrang’ (PN) and ‘Flame Seedless’ (FS) was compared; 21 discriminant metabolites were putatively annotated through chemoinformatics analysis, among which anthocyanins were significantly higher in MM, with PN contributing the anthocyanin profile and FS contributing extended shelf-life characteristics (Chatterjee et al., 2024). Comparing the metabolic profiles of hybrid offspring and their parents helps to reveal key quality-related metabolites and their metabolic pathways, providing a theoretical basis for molecular-assisted breeding (Jia et al., 2025; Li et al., 2018; Singh et al., 2024).

KG is a Euro-American hybrid tetraploid variety developed in Japan. It is sweet and juicy, has a typical strawberry aroma, and is one of the most widely cultivated table grape varieties in China. ‘Zaoheibao’ (ZHB) is a new tetraploid *Vitis vinifera* table grape variety bred by Shanxi Agricultural University by crossing the diploid ‘Guibao’ (female parent) with ‘Zaomeigui’ (male parent), followed by colchicine treatment of the hybrid seeds. It is characterized by large berries and clusters, and its most prominent feature is a strong rose aroma (Tan et al., 2017). ‘Baoguang’ (BG), ‘Cuiguang’ (CG), and ‘Miguang’ (MG) are three new table grape varieties obtained by crossing KG (female parent) with ZHB (male parent). The three offspring varieties show obvious differences in fruit color, aroma type, and sugar-acid content. In this study, using the ripe fruits of the three hybrid varieties and their parents, It was found that systematically determined basic fruit quality traits, total phenolic and anthocyanin contents, and conducted sensory evaluation. At the same time, Untargeted metabolomics based on LC-MS/MS, combined with multivariate statistical analysis and metabolic pathway enrichment analysis, was employed to compare the metabolite differences between hybrid offspring and their parents. This study aimed to reveal the genetic differences in fruit quality and metabolome among three table-grape hybrid cultivars and their parents, as well as to identify cultivar-specific metabolites and corresponding enriched metabolic pathways. Elucidating the metabolic basis for quality divergence among hybrid grape cultivars is critical for breeding cultivars with higher nutritional value, superior flavor characteristics, and improved suitability for fresh consumption, juice processing and functional-food applications.

## 2 Materials and methods

### 2.1 Test materials

#### 2.1.1 Test varieties

The BG, CG, MG, KG, and ZHB grape varieties used in this experiment were all cultivated in Jinzhong City, Shanxi Province (112.29°E, 37.20°N, altitude: 810.21 m). The region has a temperate continental climate with large diurnal temperature differences, abundant sunshine, and an effective accumulated temperature of 3610 °C. All tested varieties were grown in open fields with a plant spacing of 1.0 × 2.5 m and managed under conventional cultivation practices.

Three vines per variety (*n* = 3 biological replicates, each vine as a replicate).From each vine, 3 clusters were randomly harvested at commercial maturity (Seed development of berries reaches full maturity). From each cluster, 50 berries were randomly collected, pooled per vine, and then 100 berries per vine were used for quality and metabolomics analyses. Fig. 1 shows a flowchart of leaf SSR molecular identification and the association analysis between fruit quality traits and metabolites after fruit sampling. Harvest was performed in a single season (August 2025).

**Fig. 1:**
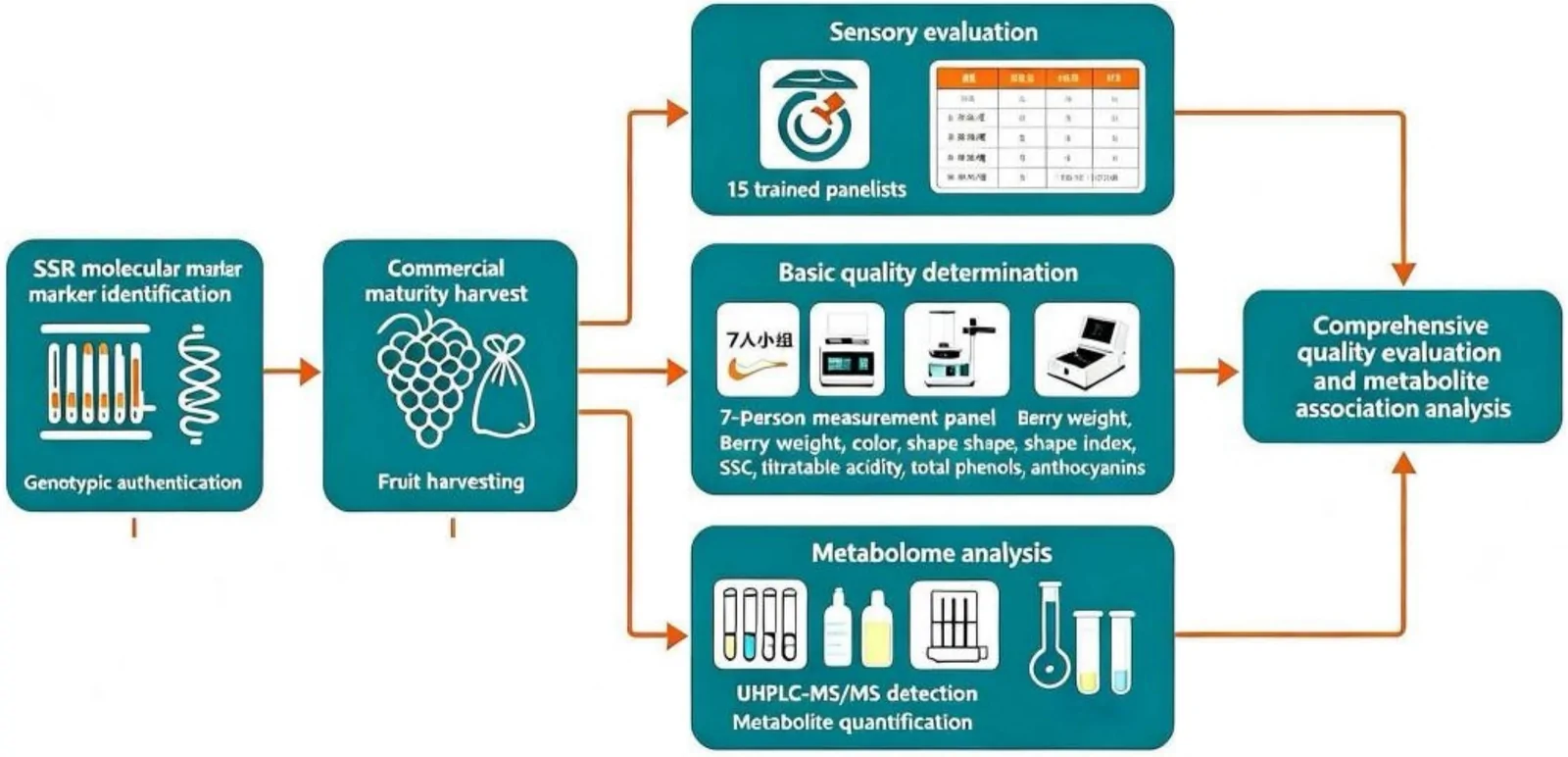
Flowchart of comprehensive fruit quality evaluation and metabolite–trait association analysis.

#### 2.1.2 Test chemicals and equipment

The chemicals, including methanol, formic acid, and ammonium acetate, were purchased from Thermo Fisher, all of LC-MS grade. The reagents used in the experiment, such as sodium hydroxide, sodium carbonate, sodium acetate, potassium chloride, and ethanol, were of analytical grade. The standard catechin was of chromatographic purity. All these reagents were produced by Sinopharm Chemical Reagent Co., Ltd. The water used in the experiment was purchased from Wahaha Group Co., Ltd. (Supplementary Table S1). The mass spectrometer adopted was Q Exactive™ HF and Q Exactive™ HF-X manufactured by Thermo Fisher (Germany), and chromatographic separation was performed on a Thermo Fisher (USA) Hypersil Gold column with specifications of 100 × 2.1 mm and a particle size of 1.9 μm. PCR instrument (Model 2720, ABI) and electrophoresis apparatus (Model DYY-8C, Beijing Liuyi Instrument Factory) were used in this study (Supplementary Table S2).

### 2.2 Experimental methods

#### 2.2.1 SSR molecular marker detection

Genomic DNA was extracted from grape leaf samples using the magnetic-bead-based method. Briefly, 27–33 mg leaf tissue was ground in liquid nitrogen, followed by lysis, RNase A treatment, water-bath incubation and centrifugation. DNA was further purified and eluted using a 96-channel nucleic acid extractor. SSR amplification was performed via a two-step PCR system with M13-tailed primers, and the Tm value was calculated as the average value of forward and reverse primers. The first-round PCR was carried out in a 10 μL reaction system with 20 amplification cycles. The product of the first-round PCR was used as the template for the second-round PCR, which was run for 30 cycles. After preliminary assessment of amplification performance by agarose gel electrophoresis, 0.3 μL PCR product was mixed with size standard and Hi-Di formamide. Following denaturation and cooling, capillary fluorescence electrophoresis was conducted on an ABI 3730xl Genetic Analyzer. The obtained raw FSA data were imported into GeneMarker 2.2.0 software. SSR fragment sizing was performed under the Fragment (Plant) mode with GS500 as the size standard, and electropherograms together with locus genotype data were exported.

#### 2.2.2 Determination of grape fruit quality

A seven-person team evaluated the fruit quality traits of the five table grape varieties according to the Descriptors and Data Standard for Grape Germplasm Resources. Single berry weight was measured by the gravimetric method, titratable acidity was determined by NaOH titration, and soluble solids content was measured using a digital refractometer.

#### 2.2.3 Determination of phenolics and anthocyanins in grape fruits

Frozen grape berries (80 berries) were taken from a − 78 °C ultra-low temperature freezer, immersed in liquid nitrogen, crushed, and the seeds were removed. The remaining material was thoroughly ground. Then, 5 g of the sample powder was transferred to a 100 mL brown volumetric flask. Three independent replicates were prepared. The sample was extracted with 70% (*v*/v) ethanol in the dark for 24 h. After filtration, the filtrate was centrifuged at 8000 ×*g* for 10 min at 4 °C, and the supernatant was collected as the phenolic extract.

Total phenolic content was determined using the Folin-Ciocalteu method. Briefly, 0.5 mL of the phenolic extract was placed in a graduated test tube, to which 1 mL of Folin-Ciocalteu reagent and 2.5 mL of 75 g/L Na₂CO₃ solution were added. The mixture was incubated in the dark at 30 °C for 40 min, and the absorbance was measured at 760 nm. Results were expressed as catechin equivalents (mg/100 g fresh weight) (Gorzin et al., 2026).

Anthocyanin content was determined using the pH-differential method. Frozen grape berries (20 berries) were taken from a − 78 °C ultra-low temperature freezer. The skins were peeled off using forceps and thoroughly ground. Then, 0.4 g of the ground skin material was weighed into a 100 mL brown volumetric flask and extracted with 1% (*v*/v) HCl in anhydrous methanol in the dark for 24 h. After filtration, the filtrate was centrifuged at 8000 ×*g* for 10 min at 4 °C, and the supernatant was collected as the anthocyanin extract. The Anthocyanin content was expressed as malvidin-3-O-glucoside equivalents per 100 g of fresh berry skin (Waterhouse et al., 2000； Kamal et al., 2026).

The pH 1.0 buffer was prepared by dissolving 1.49 g KCl in distilled water to 100 mL. Separately, 1.7 mL of concentrated HCl (analytical grade) was diluted with distilled water to 100 mL to obtain 0.2 mol/L HCl. The KCl solution and the 0.2 mol/L HCl solution were mixed at a ratio of 25:67 (*v*/v), and the pH was adjusted to 1.0 ± 0.1 with NaOH pellets.

The pH 4.5 buffer was prepared by dissolving 1.64 g sodium acetate (NaAc) in distilled water to 100 mL, and the pH was adjusted to 4.5 ± 0.1 with HCl.

For measurement, 1.0 mL of the anthocyanin extract was transferred to a 10 mL volumetric flask and made up to volume with pH 1.0 buffer, and separately with pH 4.5 buffer. After equilibration in the dark for 2 h, the absorbance of each solution was measured at 520 nm and 700 nm using a UV–vis spectrophotometer, with distilled water as the blank.

Total anthocyanin content (mg/100 g) was calculated using the following formula:

(1) $$\text{Totalanthocyanins}\left(\mathrm{mg}/100g\right)=\left(A\times \mathrm{VF}\times \mathrm{MW}\times 1,000\right)/\left(\varepsilon \times C\right)$$

(2) $$A=\left(A₅₂₀,\mathrm{pH}1.0-A₇₀₀,\mathrm{pH}1.0\right)-\left(A₅₂₀,\mathrm{pH}4.5-A₇₀₀,\mathrm{pH}4.5\right)$$

VF = dilution factor, MW = molecular weight of malvidin-3-O-glucoside (493.5 g/mol), ε = molar extinction coefficient (28,000 L·mol^−1^·cm^−1^), and C = sample weight (g).

#### 2.2.4 Sensory evaluation of grape fruits

Sensory evaluation was conducted using a professional panel consisting of 15 trained panelists.

Panel: 15 trained panelists (staff and graduate students) who passed basic taste and aroma recognition tests and received two training sessions on grape sensory attributes.

Samples: 10 berries per variety, coded with random 3-digit numbers, served at room temperature (∼22 °C) in randomised order.

Evaluation: Blind tasting, with palate cleansing (unsalted crackers and water) between samples.

Scoring: A 100-point scale with clear anchors: Appearance (30 points – color uniformity, bloom, cluster compactness), Flavor (50 points – sweetness, acidity balance, aroma intensity and typicality), Texture (20 points – firmness, juiciness, chewiness). Each panelist scored independently (Nakhaei et al., 2026).

The scoring criteria are detailed in Table 1. Each panelist independently evaluated the samples, and the results were collected for statistical analysis (Zhu, Du, et al., 2026). This study did not require ethical approval. All subjects participating in the sensory evaluation signed informed-consent and confidentiality agreements, consenting to the use of their data for research purposes. All personal information of participants was kept confidential to protect individual privacy and comply with legal standards.

**Table 1:** Sensory scoring criteria for table grapes.

Item (100 points)	Scoring criteria
Appearance (30 points)	Beautiful and well-compacted cluster, bright berry color with bloom, fresh pedicel, well-shaped berries, uniform color, fully covered with bloom
Flavor (50 points)	Excellent flavor, well-balanced sugar-to-acid ratio, possessing the characteristic aroma of grapes
Texture (20 points)	High skin turgor, easily crushed when eaten, firm and thick flesh without coarseness, juicy, few seeds

The 7-person team mentioned earlier referred to the objective fruit quality measurement team (for gravimetric, refractometer, and titration measurements); the sensory panel was the 15-person trained panel.

#### 2.2.5 Metabolite extraction from grape fruits

① Take 100 mg of liquid nitrogen-ground sample, place it into an EP tube, and add 500 μL of 80% (*v*/v) methanol aqueous solution.

② Vortex and then let stand in an ice bath for 5 min, followed by centrifugation at 15,000 *g* and 4 °C for 20 min.

③ Take an aliquot of the supernatant and dilute with LC-MS grade water to a final methanol content of 53% (*v*/v).

④ Centrifuge again at 15,000 *g* and 4 °C for 20 min, collect the supernatant, and inject into LC-MS for analysis.

#### 2.2.6 LC-MS/MS instrument parameters

UHPLC-MS/MS analyses were performed using a Vanquish UHPLC system (ThermoFisher, Germany) coupled with an Orbitrap Q ExactiveTM HF mass spectrometer or Orbitrap Q ExactiveTMHF-X mass spectrometer (Thermo Fisher, Germany) in Novogene Co., Ltd. (Beijing, China). Samples were injected onto a Hypersil Gold column (100 × 2.1 mm, 1.9 μm) using a 12-min linear gradient at a flow rate of 0.2 mL/min. The eluents for the positive and negative polarity modes were eluent A (0.1% (*v*/v) FA in Water) and eluent B (Methanol).The solvent gradient was set as follows: 2% (*v*/v) B, 1.5 min; 2–85% (*v*/v) B, 3 min; 85–100% (*v*/v) B, 10 min; 100–2% (*v*/v) B, 10.1 min； 2% (*v*/v) B, 12 min. Q ExactiveTM HF mass spectrometer was operated in positive/negative polarity mode with spray voltage of 3.5 kV, capillary temperature of 320 °C, sheath gas flow rate of 35 psi and aux gas flow rate of 10 L/min, S-lens RF level of 60, Aux gas heater temperature of 350 °C (Waterhouse et al., 2000； Kamal et al., 2026).

Sample → Vanquish UHPLC system (ThermoFisher) → Hypersil Gold column (100 × 2.1 mm, 1.9 μm) → 12 min linear gradient elution (eluent A: 0.1% (*v*/v) formic acid in water; eluent B: methanol) → Gradient programme: 2% (*v*/v) B (0–1.5 min) → 2–85% (*v*/v) B (1.5–3 min) → 85–100% (*v*/v) B (3–10 min) → 100–2% (*v*/v) B (10–10.1 min) → re-equilibration at 2% (*v*/v) B (10.1–12 min) → Orbitrap Q Exactive HF / HF-X mass spectrometer operated under positive/negative ion mode → MS/MS raw data collection.

#### 2.2.7 Data Preprocessing and metabolite identification

UHPLC-MS/MS raw data were processed using XCMS for peak detection, alignment and quantification. Peak intensities were normalized against the total spectral intensity of the first sample. Metabolite annotation was performed by matching accurate mass (10 ppm mass tolerance) and adduct information against high-quality MS/MS spectral databases to acquire qualitative and relative quantitative results.

### 2.3 Data analysis

Data analysis and plotting were performed using Excel 2019 and SAS 9.2. For fruit quality and phenolics: one-way ANOVA with variety as fixed factor, followed by Tukey's HSD post-hoc test (*p* < 0.05). Metabolomics data processing was carried out on a Linux operating system (CentOS version 6.6) using R and Python software (Chong & Xia, 2018). The performance of the random forest cultivar discrimination model was evaluated using repeated nested 10-fold cross-validation. Using the relative quantification data of all 2038 annotated metabolites, the abundance of each metabolite across samples was *Z*-score standardized to eliminate differences in abundance magnitude (Supplementary Table S4). After excluding quality control (QC) samples, a standardized matrix was constructed with samples as rows and metabolites as columns. Euclidean distances between any two samples were then calculated to generate a sample-by-sample distance matrix. The identified metabolites were annotated using the KEGG database (https://www.genome.jp/kegg/pathway.html), the HMDB database (https://hmdb.ca/metabolites), and the LIPID Maps database (http://www.lipidmaps.org/). Differential metabolites: VIP ≥ 1, |log2FC| ≥ 1, and FDR adjusted *p* < 0.05.

A weighted gene co-expression network analysis (WGCNA) was performed to construct a co-expression network using *Z*-score normalized quantitative data of all 2038 metabolites. The soft-thresholding power was determined by scale-free network topology fitting; the minimum power satisfying a scale-free topology fit index of R^2^ > 0.8 was selected as the optimal soft threshold. One-step network construction and module detection were applied, with minModuleSize set to 30 and mergeCutHeight set to 0.25. To assess module robustness, module preservation analysis was performed. The module preservation Z-summary score was calculated using 200 permutation tests; Z-summary >2 indicates good module stability, whereas Z-summary <2 suggests poor module stability.Correlations between module eigengenes and fruit quality traits were evaluated using Pearson correlation analysis. To reduce false positives arising from multiple comparisons, *P*-values were corrected using the Holm–Bonferroni method, and an adjusted FDR < 0.05 was considered statistically significant.

Prior to redundancy analysis (RDA), variance inflation factor (VIF) collinearity diagnosis was performed on explanatory variables (metabolites). Metabolites with VIF > 10 were removed to mitigate the interference of severe multicollinearity. A total of 999 permutations were used to evaluate the statistical significance of the overall RDA model and individual axes. Both raw constrained variance and adjusted constrained variance (adjusted R^2^) were reported as corrected explanatory power to eliminate over-optimistic bias in explained variance caused by the number of variables. Response variables (fruit-quality traits) were standardized.

Number of biological replicates: *n* = 3 per variety for metabolomics, *n* = 3 for fruit quality traits, *n* = 15 for sensory.

## 3 Results and analysis

### 3.1 Genetic relationship verification of parents and offspring using SSR markers

Genetic diversity and cluster analysis of three table grape varieties and their parents were conducted using 30 pairs of SSR fluorescent primers (Supplementary Table S3). As shown in Figs. 2A–2E, capillary electrophoresis analysis of the parents and three hybrids using primer VrZAG93 revealed that the three progeny possessed characteristic alleles from both the male parent ZHB and female parent KG, confirming that all three hybrids were true hybrids with genetic contributions from both parents. Among the 30 primer pairs, 28 amplified clear and reproducible bands across all five varieties, yielding a total of 28 allelic loci. A dendrogram of the three hybrids and their parents was constructed using the UPGMA method in NTSYS-pc 2.10e software (Fig. 2F). The results showed that the female parent KG and the male parent ZHB exhibited the largest genetic distance, satisfying the requirement for parental diversity in cross-breeding. The three hybrids clustered together as a single group, maintaining certain genetic distances from both parents. Based on the fingerprint data obtained from the 30 SSR loci, alleles were arranged in ascending order and assigned Arabic numerals starting from 1; failed amplifications were marked as “*”. The alleles at each locus were assigned as 1/2, 3/4, 5/6, and 7/8, respectively, yielding the concatenated string “12,345,678”. This encoded string was then converted into a unique barcode identifier using barcoding technology, thereby constructing a barcode-based molecular identity for each variety. All three hybrids and their parents generated distinct identity codes, indicating clear genetic differentiation among the five varieties.

**Fig. 2:**
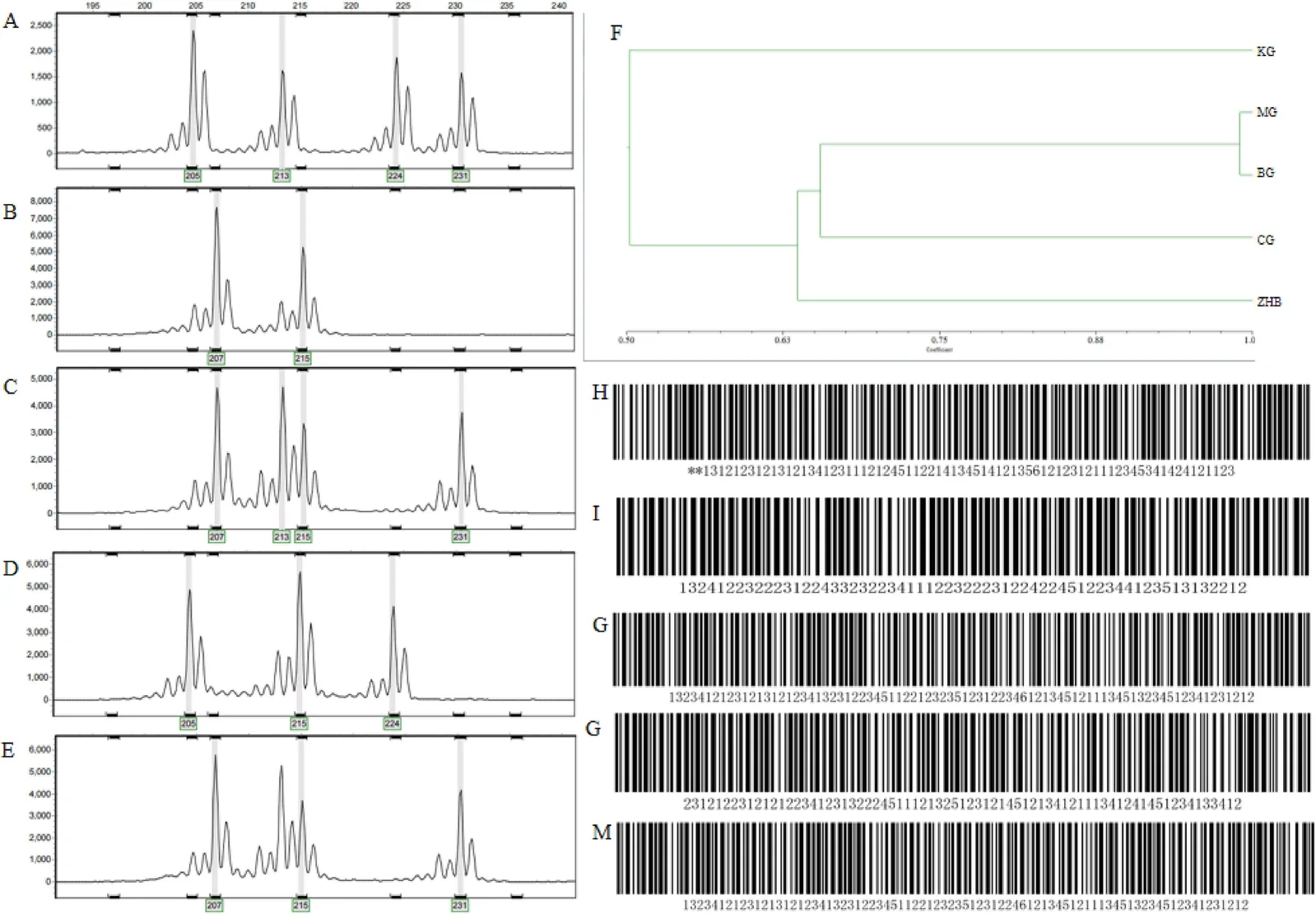
Genetic diversity analysis of three table grape varieties and their parents based on 30 pairs of SSR fluorescent primers. Note: Figs. 2A–2E represent the capillary electrophoresis profiles of KG, ZHB, BG, CG, and MG, respectively, obtained with primer VrZAG93; Fig. 2F is the UPGMA dendrogram; Figs. 2H–2M represent the barcode-based molecular identity cards of KG, ZHB, BG, CG, and MG, respectively.

### 3.2 Comparison of basic fruit quality among three table grape varieties and their parents

The fruit quality traits of the five grape varieties were measured. As shown in Table 2, the average berry weights of CG and ZHB were significantly higher than those of the other varieties, at 9.16 g and 9.07 g, respectively. The fruit shape indices of BG and KG were close to 1, indicating nearly round berries. Soluble solids content was highest in CG (19.26%) and KG (19.06%). Moreover, CG had the lowest titratable acid content at 3.78 g·L^−1^, demonstrating excellent sugar-acid balance with high sugar and low acidity. Regarding aroma, MG resembled its male parent ZHB, featuring a strong rose aroma as the main characteristic. CG and KG exhibited strawberry aromas, while BG possessed both light rose and light strawberry aromas.

**Table 2:** Basic fruit quality of three table grape varieties and their parents.

Variety	Average fruit weight/g	Skin color	Fruit shape index	Soluble solids/%	Titratable acid content/g·L-1	sugar-acid ratio	Aroma
BG	8.28 ± 0.78ab	purple-black	1.06 ± 0.02c	18.36 ± 0.78a	4.12 ± 0.11c	44.56 ± 1.22b	light rose and light strawberry aroma
CG	9.16 ± 0.46a	purple-black	1.16 ± 0.01b	19.26 ± 1.07a	3.78 ± 0.17d	50.95 ± 2.16a	light strawberry aroma
MG	7.96 ± 0.83b	purple-red	1.12 ± 0.03b	17.05 ± 0.84b	4.52 ± 0.09b	37.72 ± 2.44d	rose aroma
KG	7.42 ± 0.44b	purple-black	1.04 ± 0.02c	19.06 ± 0.67a	5.18 ± 0.24a	36.80 ± 2.42d	strawberry aroma
ZHB	9.07 ± 0.62a	purple-red	1.27 ± 0.02a	17.57 ± 0.88b	4.16 ± 0.16c	42.24 ± 1.03c	strong rose aroma

Note: Values are mean ± SD (*n* = 3); different lowercase letters indicate significant differences among varieties (one-way ANOVA, Tukey's HSD, *p* < 0.05), whereas *p* > 0.05 was considered “not significant.”

### 3.3 Comparison of phenolics and anthocyanins among three table grape varieties and their parents

Phenolics and anthocyanins are core functional active components in grapes, possessing strong antioxidant activity. The total phenolic and anthocyanin contents in the fruits of the three table grape varieties and their parents are shown in Fig. 3, with significant differences observed among varieties. The total phenolic content was highest in the female parent KG (14.26 mg/100 g), and MG showed no significant difference from the female parent KG, while CG had the lowest total phenolic content. BG exhibited the highest anthocyanin content in the skin (14.56 mg/100 g), which was significantly higher than that of the male parent ZHB. KG was characterized by high total phenolic content with moderate anthocyanin content, BG was distinguished by high anthocyanin content, and CG showed relatively high anthocyanin content but low total phenolic level. The varieties also exhibited certain differences in antioxidant capacity, skin coloration, and flavor formation.

**Fig. 3:**
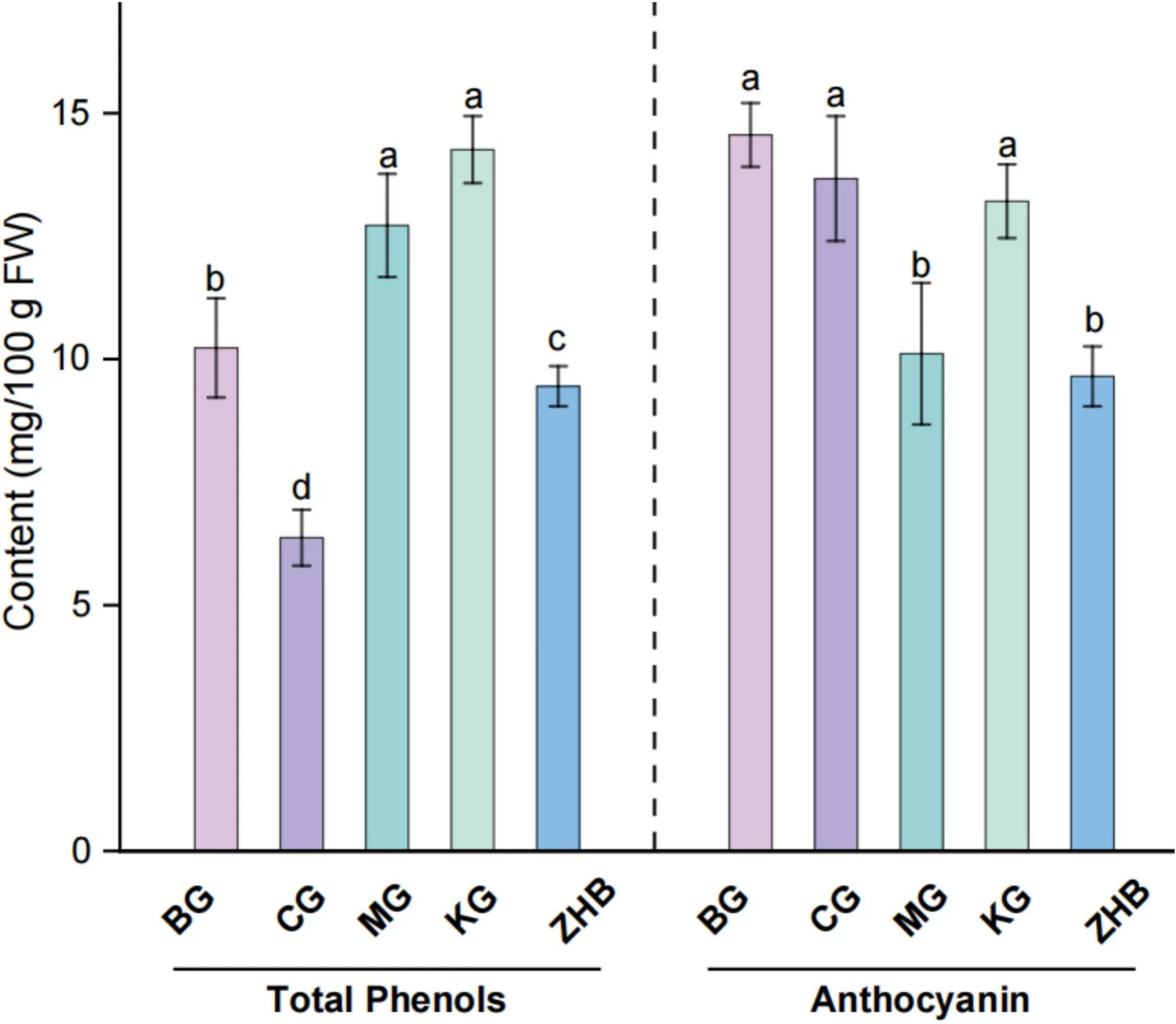
Total phenolic and anthocyanin contents in fruits of three table grape varieties and their parents. Note: Error bars represent standard deviation (SD), n = 3. Different lowercase letters indicate significant differences among varieties (one-way ANOVA, Tukey's HSD, p < 0.05), whereas p > 0.05 was considered “not significant.”

### 3.4 Sensory evaluation of three table grape varieties and their parents

The sensory evaluation results based on three dimensions (appearance, flavor, and texture) are shown in Fig. 4. There were significant differences in the overall sensory quality among the different grape varieties. Among them, MG had the highest comprehensive score, excelling in appearance, flavor, and texture. It featured a well-balanced sugar-to-acid ratio, rich aroma, firm and thick flesh, and good juiciness, meeting the evaluation criteria for high-quality table grapes. BG and ZHB had moderately high comprehensive sensory quality, with good appearance uniformity and flavor performance. CG had a relatively low sensory score, mainly due to its cluster appearance and flavor intensity; however, CG scored high in texture, exhibiting crisp flesh. The sensory evaluation indicated that MG had the most prominent advantages in flavor and texture.

**Fig. 4:**
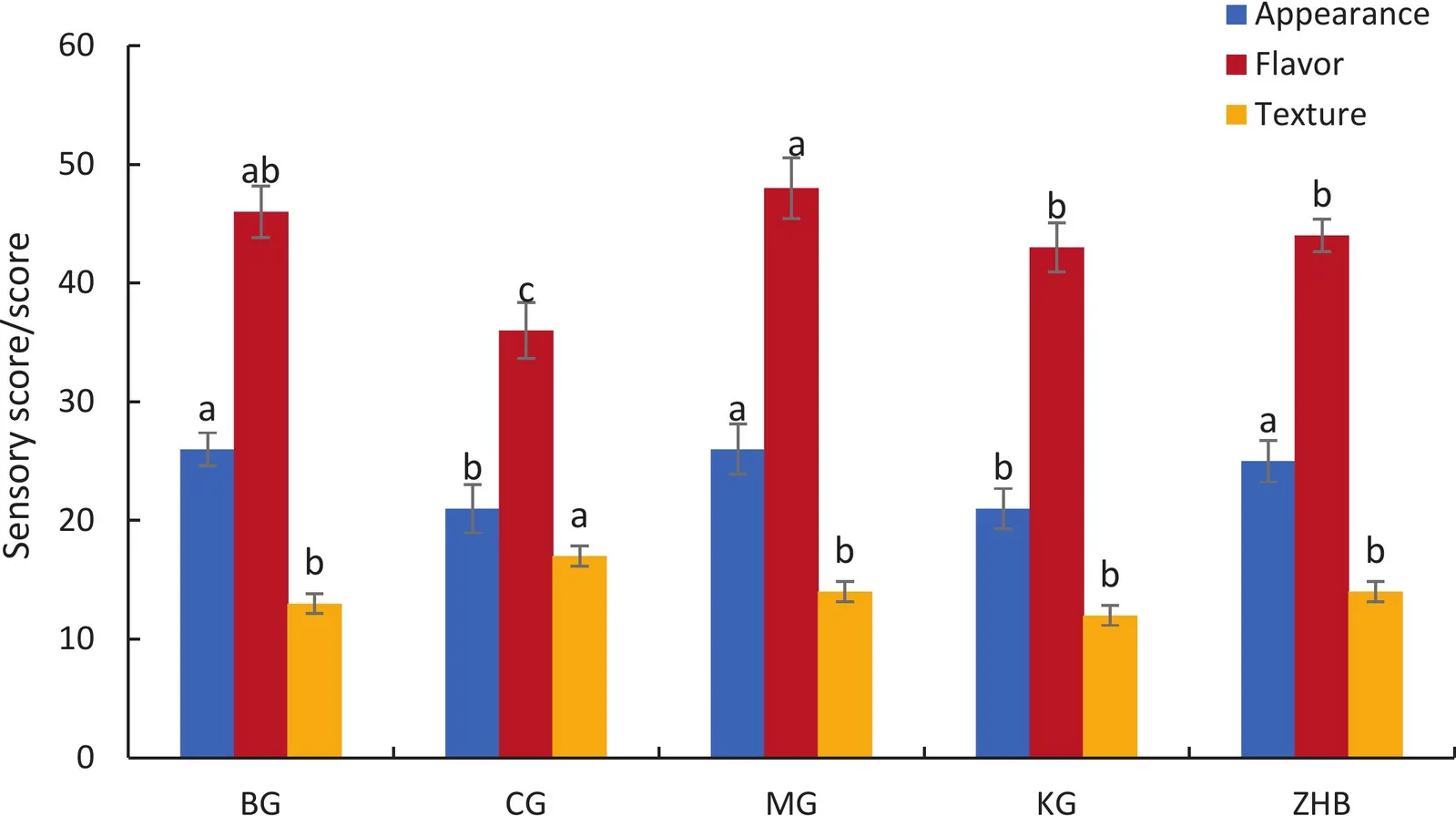
Sensory evaluation of three table grape varieties and their parents. Note: Error bars represent standard deviation (SD), n = 15. Different lowercase letters indicate significant differences among varieties (one-way ANOVA, Tukey's HSD, p < 0.05), whereas p > 0.05 was considered “not significant.”

### 3.5 Metabolomics analysis of three table grape varieties and their parents

#### 3.5.1 Classification of metabolites among three table grape varieties and their parents

Model performance assessed via repeated nested 10-fold cross-validation (5 repeats) yielded an mean outer-loop cross-validation accuracy of 97.42 ± 0.86% (mean ± SD). The ordinary single-run 10-fold cross-validation gave 98.7% accuracy (Fig. 5A). nested repeated cross-validation produced a slightly lower but more realistic estimate of generalization performance. The confusion matrix revealed minor classification uncertainty for individual samples. The metabolome-based model can discriminate the five tested cultivars well, but its performance cannot be guaranteed for other external grape varieties.

**Fig. 5:**
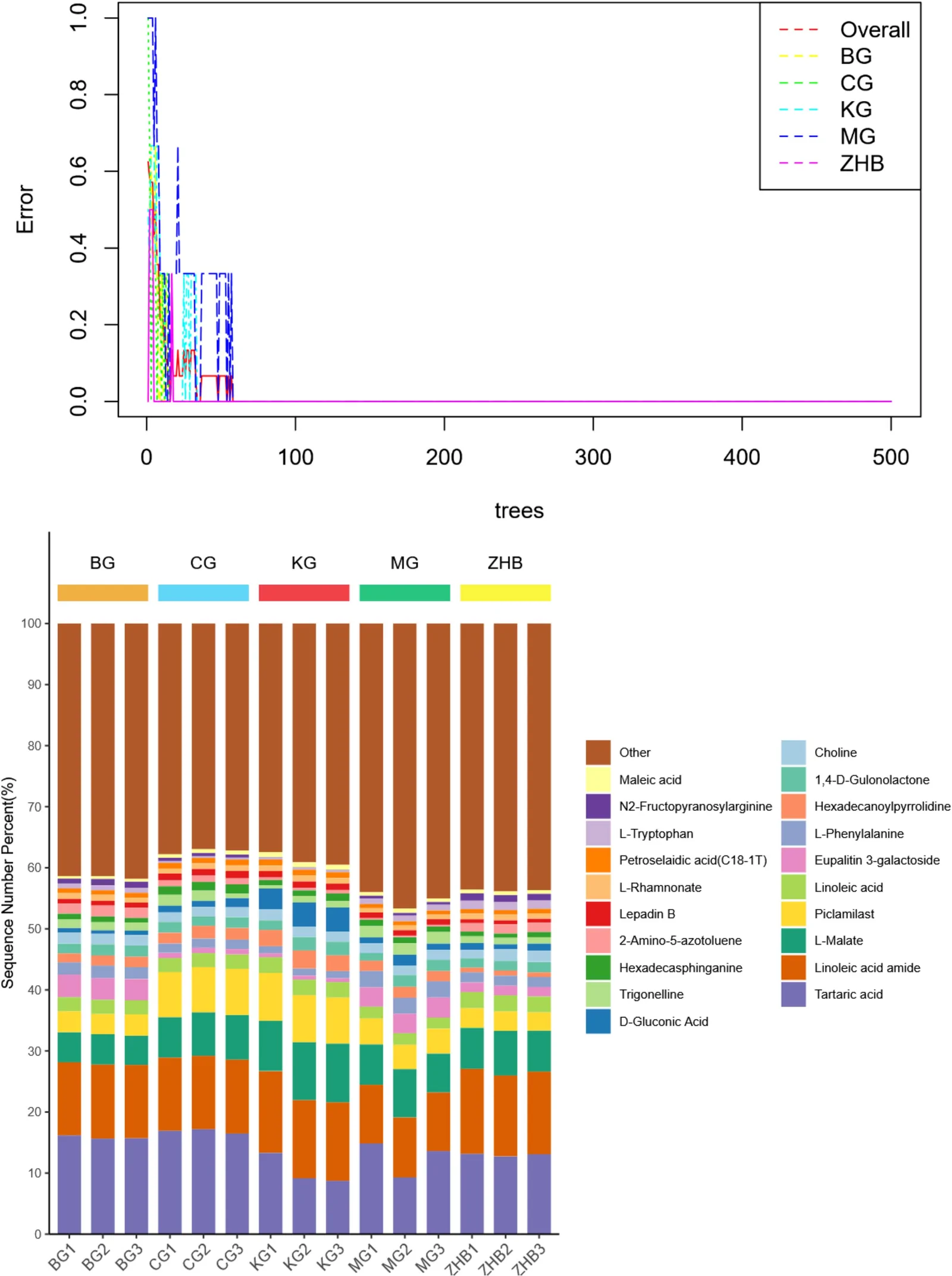
Metabolites of three table grape varieties and their parents. Note: Fig. 5A: Random forest classification model; Fig. 5B: Classification of metabolite categories.

Differences in metabolite categories among the three table grape varieties and their parents are shown in Fig. 5B. The metabolic profiles of biological replicates of the same variety showed high consistency, indicating good experimental reproducibility. The metabolite compositions of the five grape varieties were significantly different. All three hybrid varieties exhibited a superposition of metabolic traits from both parents. Among them, the proportion of *L*-phenylalanine in BG fruits was significantly higher than that in both parents; CG showed significantly higher proportions of L-malic acid and D-gluconic acid; MG had the highest proportion of L-rhamnose acid and the lowest proportion of lipids. The differences in metabolic profiles between hybrid offspring and their parents were mainly reflected in the proportions of aromatic amino acids, organic acids, and lipids, which may be closely related to the aroma, flavor, and quality characteristics of the different varieties.

#### 3.5.2 PCA and OPLS-DA analysis of metabolites among three table grape varieties and their parents

The OPLS-DA model was validated by permutation testing (*n* = 200). The Q^2^ intercept was −0.5224, well below zero, confirming no overfitting. The *p*-values for both R^2^Y and Q^2^ were *p* = 0.005, demonstrating the statistical significance and good predictive ability of the model. (Fig. S1). PCA and OPLS-DA analysis of metabolites among the table grape varieties and their parents are shown in Fig. 6. Fig. 6A shows the PCA score plot of the five varieties. PC1 and PC2 explained 33.9% and 20.3% of the total variance, respectively, with a cumulative explanation rate of 54.2%, effectively reflecting the metabolomic differences among samples. Clear inter-group separation was observed among different varieties. The metabolic profiles of the three table grape varieties were significantly different from those of their parents, and the three hybrid varieties also formed independent clusters, indicating that the hybrid offspring have developed variety-specific metabolomic characteristics distinct from their parents. To screen for key differential metabolites distinguishing the grape varieties, VIP analysis was performed based on the OPLS-DA model. Fig. 6B shows Combined VIP versus significance (−log₁₀ FDR adjusted p) scatter plot of key differential metabolites in the OPLS-DA model. Metabolites with VIP ≥ 1 and FDR-adjusted *p* < 0.05 were defined as differentially accumulated metabolites. A large number of key metabolites contributing significantly to inter-variety differences were putatively annotated, mainly including flavonoids (e.g., Isorhamnetin 3-(6″-acetylglucoside), Peonidin-3-O-galactoside), phenolics, amino acids (e.g., L-Methionine), terpenoids, and alkaloids (e.g., BRUCEOLIDE, Cassiaside). Among them, flavonoids and anthocyanins accounted for the highest proportion of key differential metabolites, indicating that the metabolomic differences among grape varieties are mainly reflected in the flavonoid and anthocyanin biosynthesis pathways. These differential metabolites may be closely related to the color, flavor, and antioxidant properties of grape berries.

**Fig. 6:**
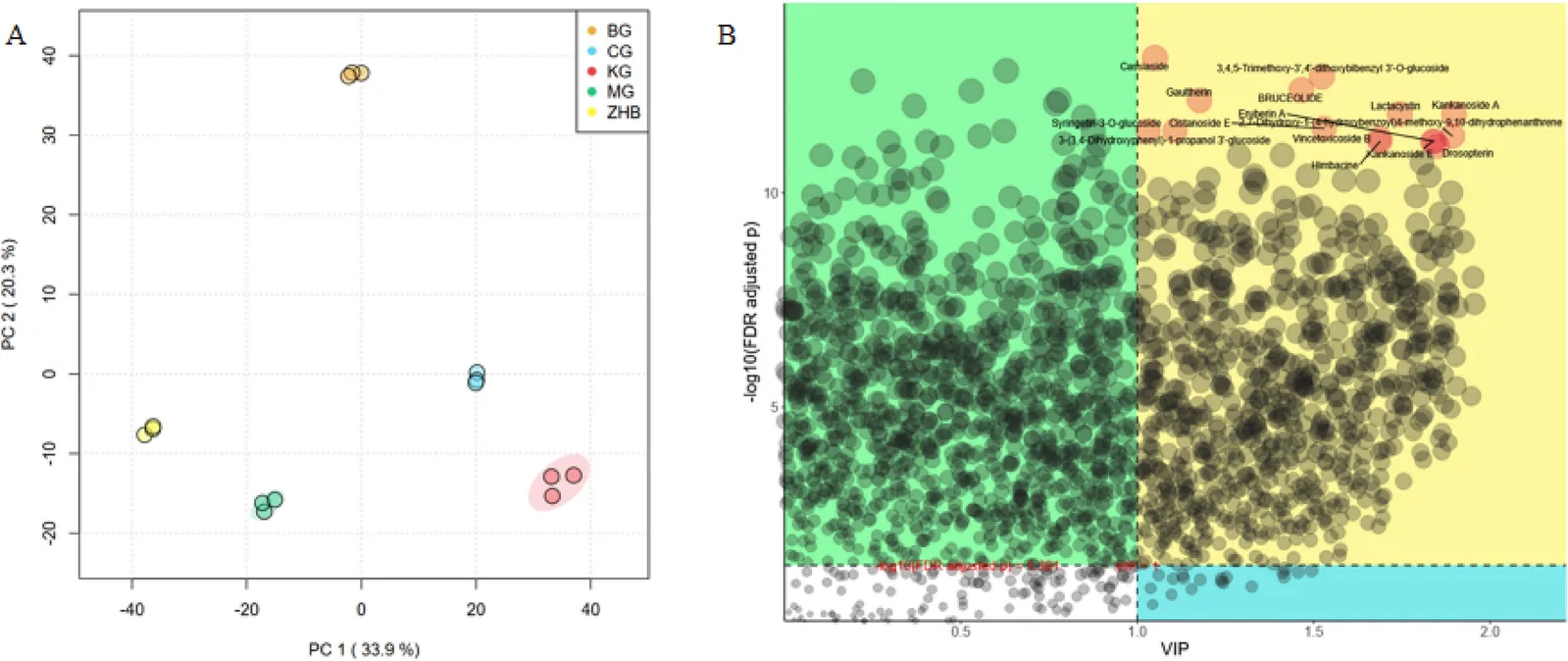
PCA and OPLS-DA analysis of metabolites among three table grape varieties and their parents. Note: Fig. 6A: PCA score plot; Fig. 6B: Combined VIP versus significance (−log₁₀ FDR adjusted p) scatter plot of key differential metabolites in the OPLS-DA model.

#### 3.5.3 Comparative analysis of differential metabolites among three table grape varieties and their parents

To elucidate the differences in metabolites between hybrid offspring and their parents, volcano plots comparing the metabolites of the three table grape varieties with their parents are shown in Fig. 7. Fig. 7A shows the comparison between BG and the female parent KG. Differential metabolites were screened based on VIP ≥ 1, |log₂FC| ≥ 1, and FDR-adjusted p (q) < 0.05.Among the 2038 putatively annotated metabolites, 367 were significantly upregulated and 331 were significantly downregulated, with the number of upregulated metabolites being significantly higher than that of downregulated ones. A large number of flavonoids, aromatics, and amino acids (e.g., L-Methionine, Isorhamnetin 3-(6″-acetylglucoside)) were specifically upregulated in BG, forming a unique metabolic profile characterized by high expression of flavonoids and aroma precursors. Compared with the male parent ZHB (Fig. 7B), BG had 393 significantly upregulated metabolites and 301 significantly downregulated metabolites. The metabolome of BG inherited some traits from both parents while exhibiting significant activation of secondary metabolism, showing transgressive advantage and undergoing substantial reorganization to form a unique metabolic profile distinct from both parents.

**Fig. 7:**
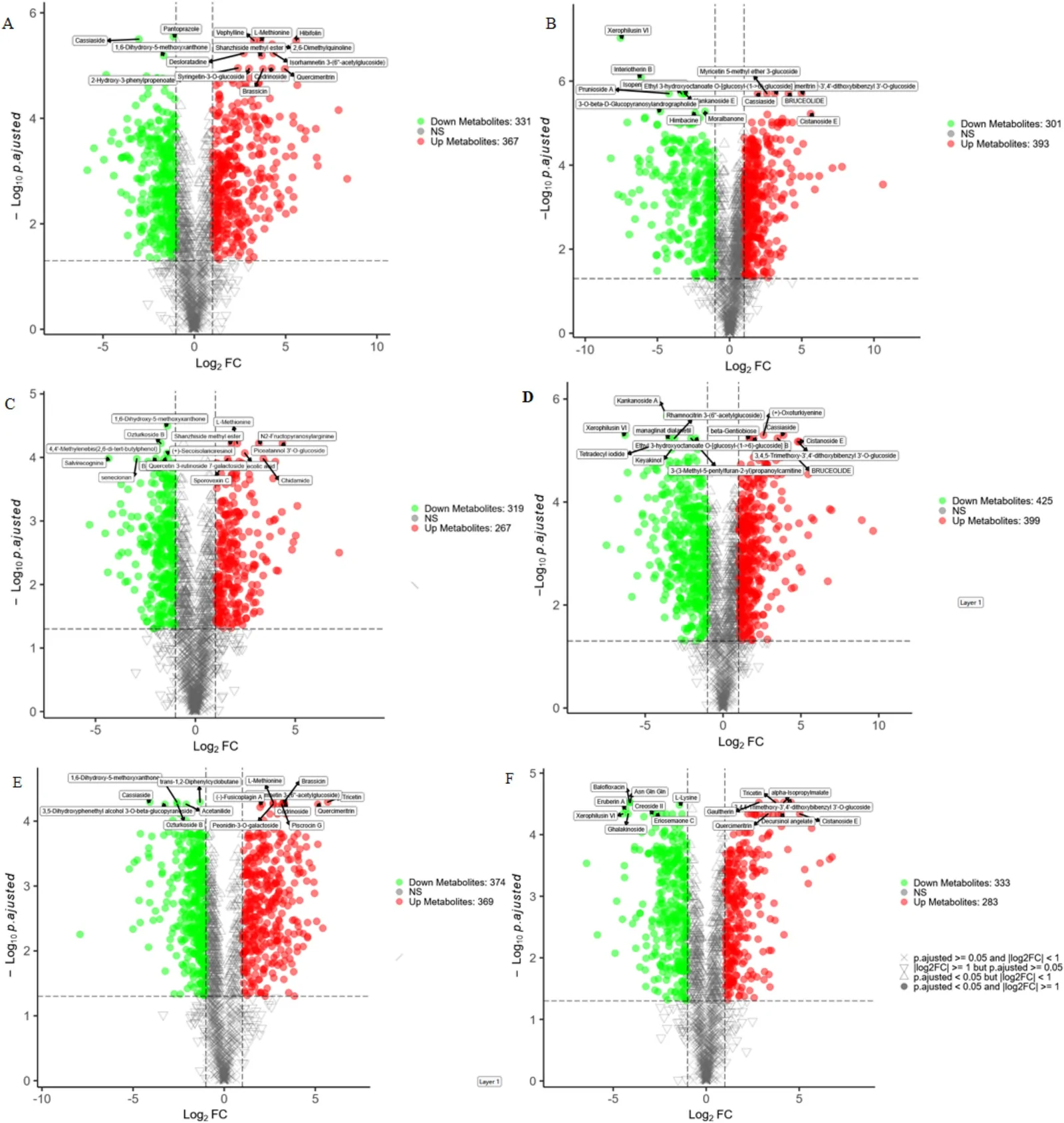
Volcano plots comparing metabolites of three table grape varieties with their parents. Note: Fig. 7A: BG vs. KG; Fig. 7B: BG vs. ZHB; Fig. 7C: CG vs. KG; Fig. 7D: CG vs. ZHB; Fig. 7E: MG vs. KG; Fig. 7F: MG vs. ZHB.

As shown in Fig. 7C, comparing CG with the female parent KG, a total of 319 significantly downregulated metabolites and 267 significantly upregulated metabolites were putatively annotated. Metabolites such as flavonoids and aromatic amino acids (e.g., L-Methionine) were highly expressed in CG fruits. Compared with the male parent ZHB (Fig. 7D), CG had 425 significantly downregulated metabolites and 399 significantly upregulated metabolites. The metabolic profile of CG significantly favored the female parent, forming a metabolic pattern closer to KG.

As shown in Fig. 7E, comparing MG with the female parent KG, MG fruits had 374 significantly downregulated metabolites and 369 significantly upregulated metabolites. Metabolites such as L-Methionine, Peonidin-3-O-galactoside, and Quercimeritrin (flavonoid glycosides, anthocyanins, and aromatic amino acids) were specifically upregulated in MG. Compared with the male parent ZHB (Fig. 7F), MG had 333 significantly downregulated metabolites and 283 significantly upregulated metabolites. Some secondary metabolites highly expressed in ZHB were significantly reduced, while a group of new flavonoid glycosides and phenolic derivatives were specifically upregulated. The metabolome of MG underwent significant reprogramming during hybridization, exhibiting marked activation of secondary metabolic pathways, forming a unique metabolic pattern distinct from both parents, which is closely related to its strong rose aroma and excellent fruit quality.

The differences in metabolites between the three hybrid varieties and their parents varied depending on the variety, showing different upregulation and downregulation biases. Rather than simply favoring one parent, the hybrids formed variety-specific metabolic profiles through hybridization, exhibiting distinct inheritance and remodeling of metabolic traits from both parents.

#### 3.5.4 Cluster heatmap analysis of differential metabolites among three table grape varieties and their parents

Based on the relative quantification data of all 2038 annotated metabolites, Euclidean distances between samples were calculated after *Z*-score standardization, yielding the intra- and inter-variety distance matrix (Table 3). The diagonal values represent the average distances among biological replicates within each variety, ranging from 17.85 to 21.46, indicating good consistency of metabolic profiles among replicate samples of the same variety. The inter-variety Euclidean distances ranged from 62.17 to 77.70, with the largest distance observed between the female parent KG and the male parent ZHB (77.70), indicating the most significant metabolomic difference between the two parents. Among the hybrids, CG showed the smallest distance to the female parent KG (62.17), supporting the conclusion that CG has a higher metabolomic similarity to KG. The distance between MG and the male parent ZHB (66.97) was slightly smaller than that between MG and the female parent KG (67.54), indicating a slightly higher metabolomic similarity of MG to the male parent ZHB. BG exhibited relatively large distances to all varieties (63.65–70.65) and was far from both parents, suggesting that it formed a unique metabolic profile.

**Table 3:** Euclidean Distance Matrix of Three Cultivated Grape Varieties and Their Parents.

	BG	CG	MG	KG	ZHB
BG	18.11	65.11	63.65	70.65	67.84
CG		17.85	62.62	62.17	73.44
MG			20.21	67.54	66.97
KG				21.46	77.70
ZHB					18.30

To further analyze the expression patterns of differential metabolites among the different varieties, cluster heatmap analysis was performed on the top 30 metabolites with the highest VIP values from the OPLS-DA analysis. As shown in Fig. 8, the heatmap of characteristic differential metabolites of the three table grape varieties and their parents revealed two major branches based on metabolic profile similarity: MG clustered with the male parent ZHB, and both exhibited high expression levels of flavonoid glycosides, phenolic acids, and terpenoid derivatives (e.g., Tricalysioside R, Kankanoside A). MG significantly overexpressed a large number of characteristic secondary metabolites of the male parent ZHB, showing greater metabolomic similarity to the male parent. CG clustered with the female parent KG, and the metabolic profile of CG was closer to that of KG, showing greater metabolomic similarity to the female parent. Phenolic and organic acid metabolites such as Holyryne A, 10-Hydroxydecanoate, and Difucol hexaacetate were highly expressed in BG, representing unique metabolic features that distinguish BG from the other varieties.

**Fig. 8:**
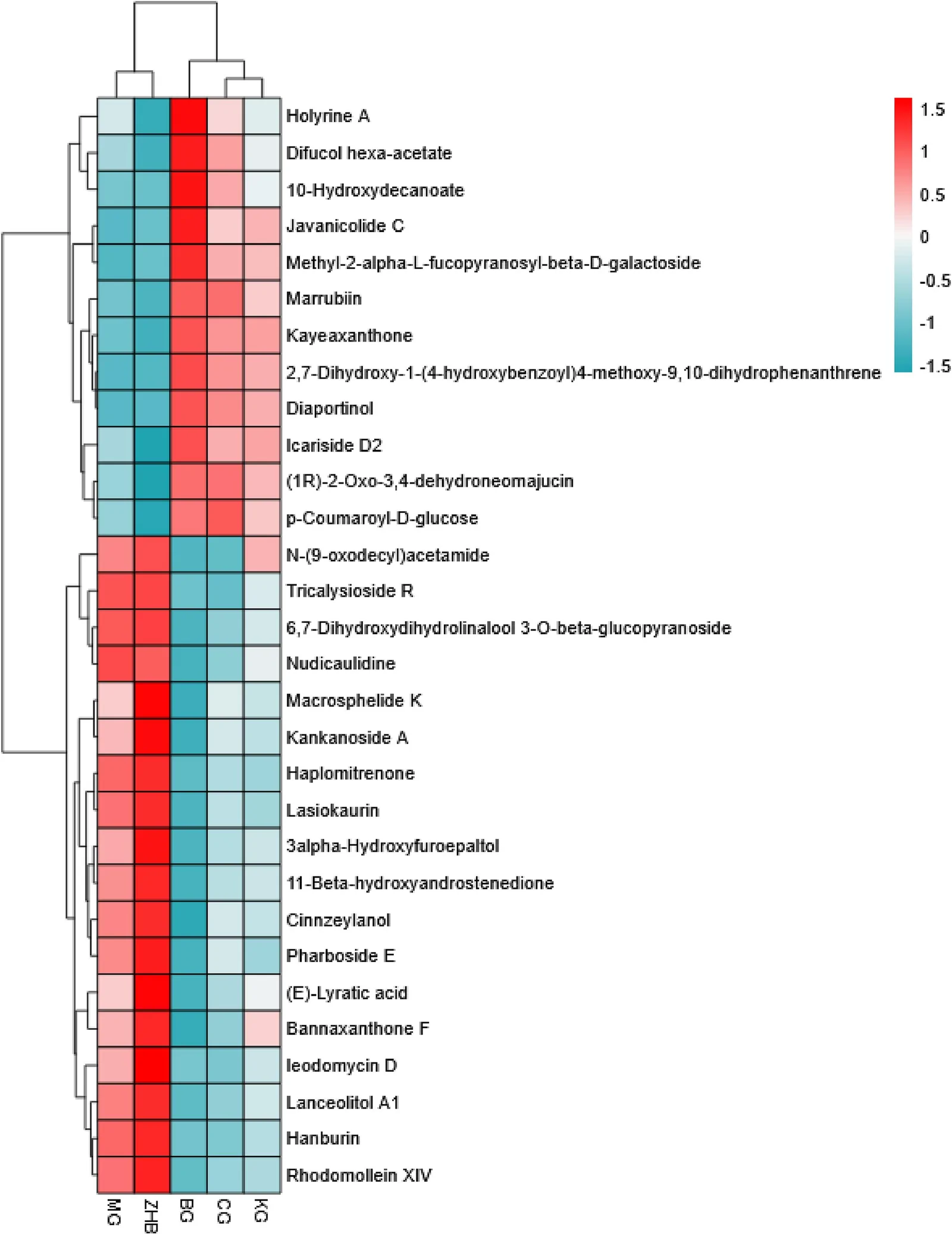
Cluster heatmap of differential metabolites among three table grape varieties and their parents.

#### 3.5.5 KEGG pathway enrichment analysis of differential metabolites among three table grape varieties and their parents

To analyze the functional distribution of differential metabolites between the three table grape varieties and their parents, KEGG pathway enrichment analysis was performed. As shown in Fig. 9, the KEGG enrichment results for the three varieties compared with their parents are presented.The input dataset for enrichment analysis consisted of the significantly differential metabolites identified from volcano plot analysis (|log₂FC| ≥ 1, VIP ≥ 1, and FDR-adjusted *p* < 0.05). Fig. 9A shows the enrichment analysis for BG vs. the female parent KG. The differential metabolites were mainly enriched in pathways related to amino acid metabolism and plant secondary metabolism. Among them, the “Biosynthesis of amino acids” pathway showed the highest enrichment level and contained the largest number of differential metabolites, making it the most significantly enriched pathway, followed by “Biosynthesis of various plant secondary metabolites”, “Flavone and flavonol biosynthesis”, and “Aminoacyl-tRNA biosynthesis”. Fig. 9B shows the enrichment analysis for BG vs. the male parent ZHB. The differential metabolites were mainly enriched in flavonoid-related secondary metabolic pathways, among which “Flavone and flavonol biosynthesis” had the highest enrichment level, followed by “Flavonoid biosynthesis”, “Vitamin B6 metabolism”, and “Zeatin biosynthesis”. The metabolic differences between BG and both parents were primarily driven by global remodeling of the amino acid metabolism network, which directly affected the flavonoid secondary metabolic pathway. This is the key molecular basis for the differences in fruit flavor, color, and antioxidant properties between BG and its parents.

**Fig. 9:**
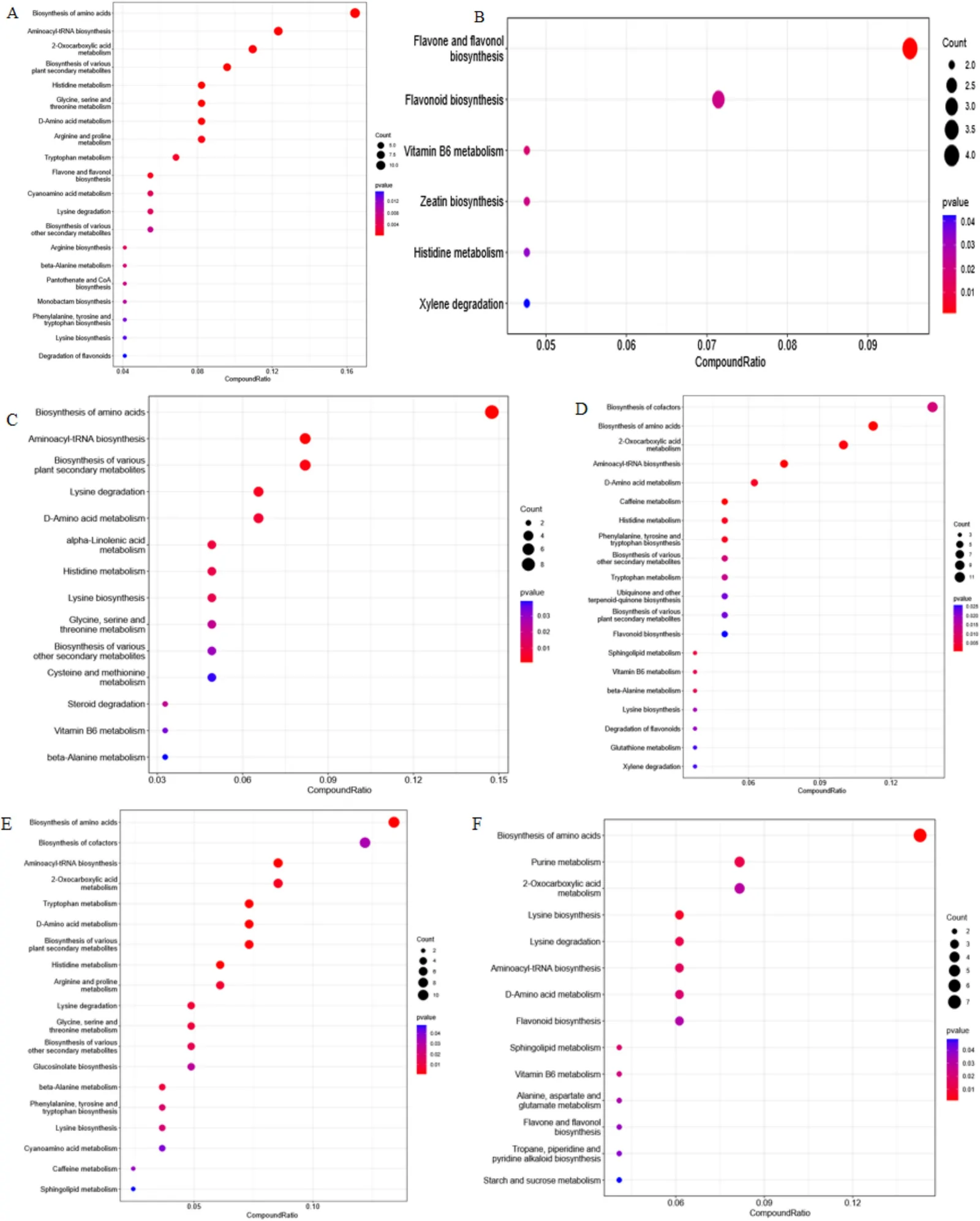

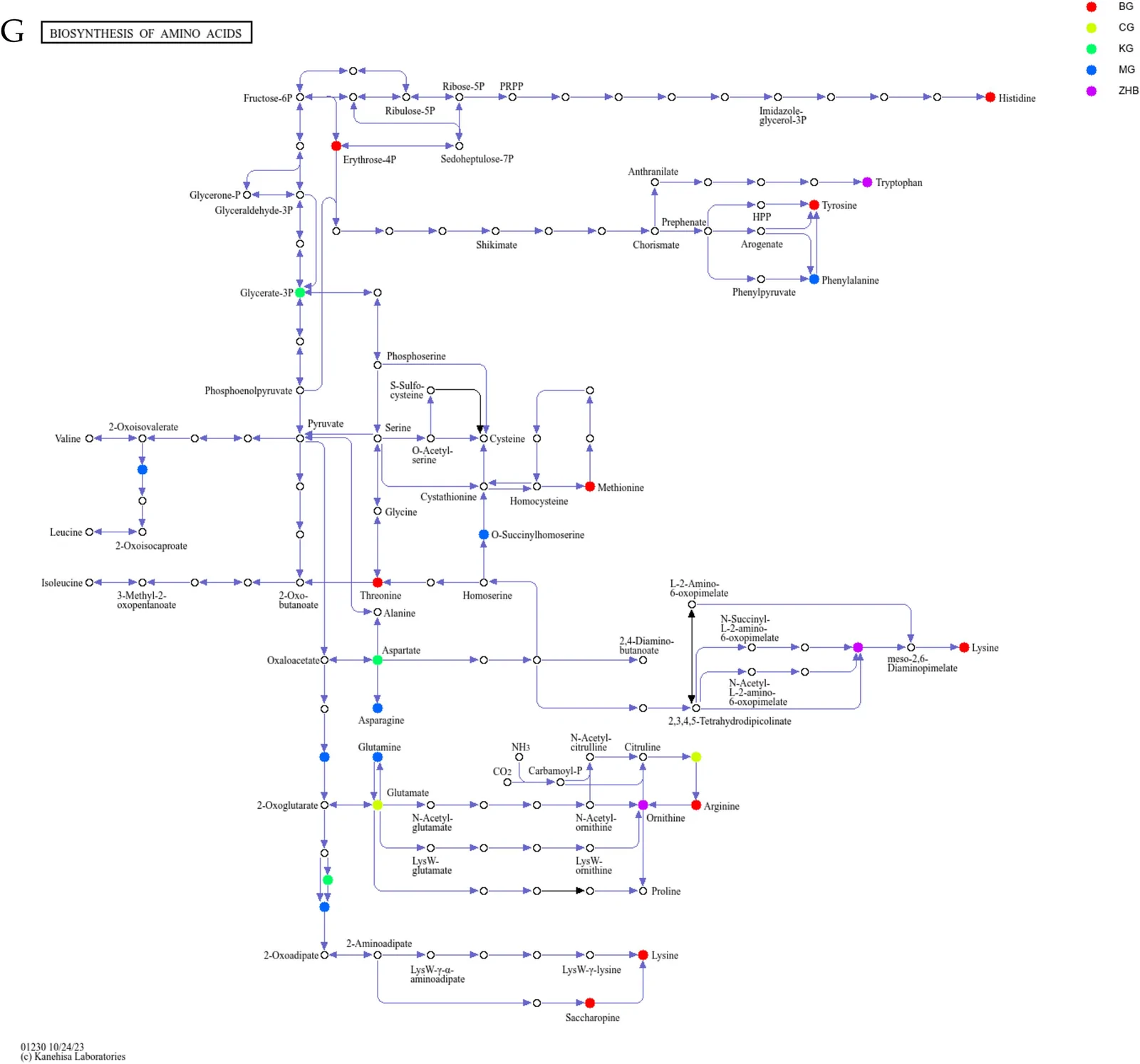
KEGG enrichment map of differential metabolites among three table grape varieties and their parents. Note: Fig. 9A: BG vs. KG; Fig. 9B: BG vs. ZHB; Fig. 9C: CG vs. KG; Fig. 9D: CG vs. ZHB; Fig. 9E: MG vs. KG; Fig. 9F: MG vs. ZHB. Fig. 9 G: KEGG pathway map of amino acid biosynthesis (ko01230). KEGG enrichment analysis was performed using the significantly differential metabolites identified from the volcano plot analysis (|log₂FC| ≥ 1, VIP ≥ 1, and FDR-adjusted q < 0.05).

Fig. 9C shows the enrichment analysis for CG vs. the female parent KG. The differential metabolites were mainly enriched in pathways related to amino acid metabolism and plant secondary metabolism. Among them, the “Biosynthesis of amino acids” pathway showed the highest enrichment level, followed by “Biosynthesis of various plant secondary metabolites”, “Aminoacyl-tRNA biosynthesis”, and “alpha-Linolenic acid metabolism”. The metabolic differences between CG and KG were associated with their quality differentiation. Which in turn affects secondary metabolism and aroma compound synthesis pathways. This may be the key molecular basis for the differences in fruit flavor and quality between the two. Fig. 9D shows the enrichment analysis for CG vs. the male parent ZHB. The differential metabolites were mainly enriched in pathways related to cofactor biosynthesis and amino acid metabolism. The metabolic differences between CG and its parents are mainly driven by global remodeling of cofactor metabolism and the amino acid metabolism network, which in turn affects secondary metabolism such as flavonoid synthesis and aroma compound synthesis pathways.

Fig. 9E shows the enrichment analysis for MG vs. the female parent KG, and Fig. 9F shows the enrichment analysis for MG vs. the male parent ZHB. For MG compared with both parents, the “Biosynthesis of amino acids” pathway consistently showed the highest enrichment level. The metabolic differences between MG and its parents are mainly driven by global remodeling of the amino acid metabolism network. Enhanced purine metabolism provides energy support for amino acid synthesis, and alterations in cofactor metabolism provide an enzymatic basis for this process, further influencing secondary metabolic pathways. This may be the key molecular basis for MG's heterosis (transgressive advantage) and its formation of unique flavor and quality.

The amino acid biosynthesis pathway (ko01230) was the most significantly enriched pathway in all hybrid–parent comparisons. This pathway integrates the biosynthetic routes of all 20 proteinogenic amino acids and serves as a hub linking primary carbon/nitrogen metabolism to secondary metabolism. Pathway mapping (Fig. 9G) revealed that *L*-phenylalanine, the entry substrate of the phenylpropanoid pathway, was relatively abundant in BG, consistent with its enhanced anthocyanin accumulation (14.56 mg/100 g). In CG, the elevation of L-malic acid, a key organic acid in grape berries, the sugar-acid ratio of CG is significantly higher than those of the other cultivars. Collectively, these findings indicate that hybridization induced differential remodeling of the amino acid biosynthetic network, which in turn modulated the flux toward downstream secondary metabolic pathways (e.g., flavonoid biosynthesis, ko00941), ultimately shaping the distinct color, flavor, and nutritional profiles of the three hybrid cultivars.

### 3.6 Redundancy analysis (RDA) reveals the metabolite–trait–variety association landscape

To preliminarily screen candidate metabolites directly associated with fruit quality traits, correlation analysis was performed using 30 key differential metabolites (VIP > 1 and |log₂FC| ≥ 1 from OPLS-DA) as the independent variables (X) and 9 major quality traits as the dependent variables (Y) (Fig. 10A). At a threshold of |r| > 0.6, flavonoids and their glycosides showed strong positive correlations with anthocyanin content (*r* = 0.69–0.78) and total phenolic content (*r* = 0.65–0.74), indicating that these metabolites are important contributors to inter-varietal differences in pigments and phenolics. Notably, DENTICULAFLAVONOL exhibited positive correlations with anthocyanins, total phenolics, and flavonoids simultaneously (all *r* > 0.65), suggesting that it may serve as a hub metabolite in the anthocyanin–phenolic metabolic network. This result is consistent with the highest anthocyanin content observed in BG. Furthermore, 12-hydroxyjasmonic acid and Threonic acid were positively correlated with titratable acidity, and their accumulation patterns in CG may be associated with its lowest titratable acidity. LysoPE and Glucosyl-C18-sphingosine were positively correlated with texture scores. These metabolites are involved in cell membrane composition or cell wall metabolism, and their association with texture may provide a molecular basis for the “crisp flesh” trait of CG. Octyl 2-furoate was positively correlated with flavor scores; furan esters typically possess fruity aromas, suggesting its potential contribution to the rose aroma of MG.

**Fig. 10:**
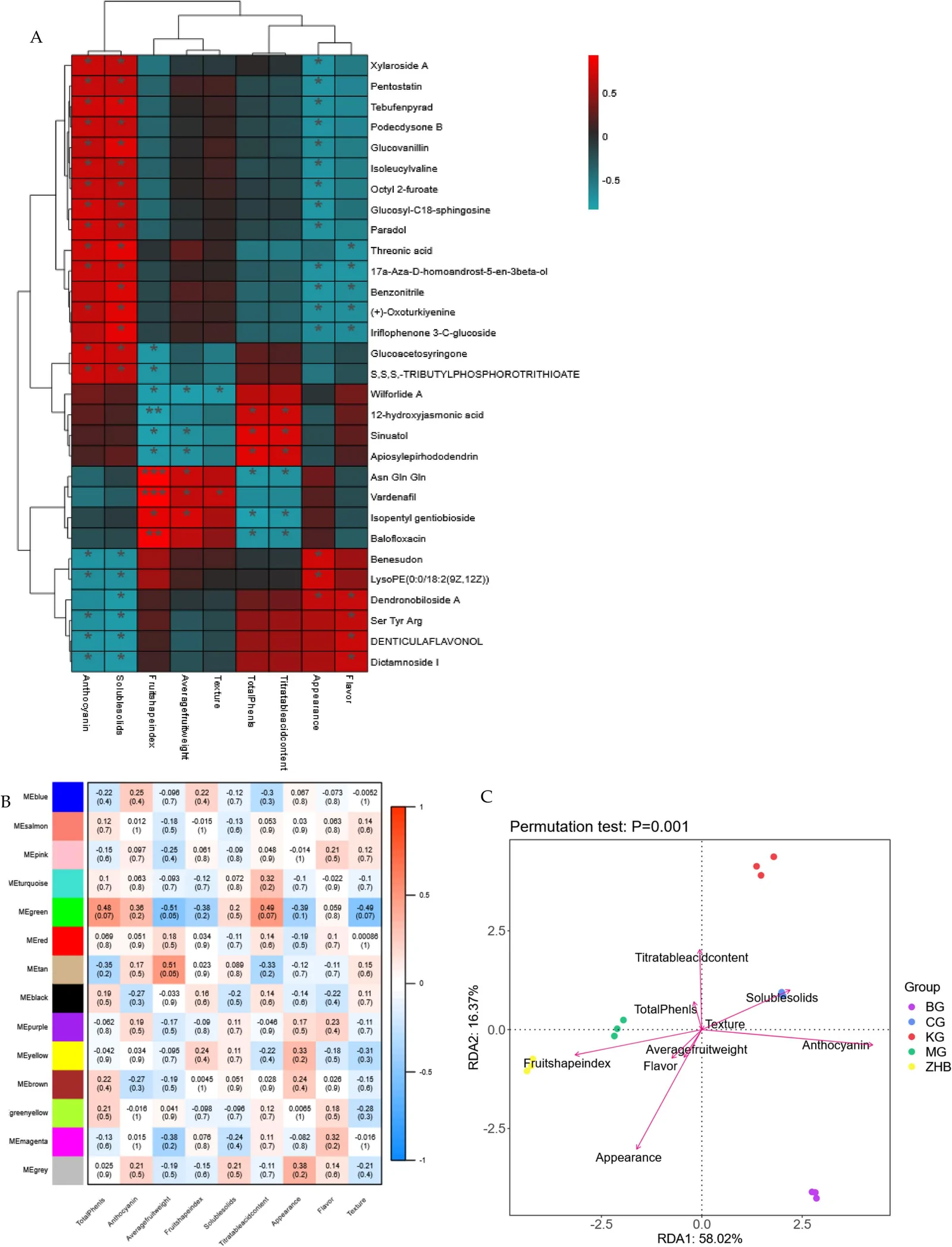
Multi-method integrative analysis of metabolite–trait associations. Note: Fig. 10A: Spearman correlation heatmap between key differential metabolites (VIP > 1 and |log₂FC| ≥ 1) and major quality traits. Red and blue indicate positive and negative correlations, respectively, with color intensity reflecting the magnitude of the correlation coefficient. *p < 0.05, **p < 0.01; Fig. 10B: Heatmap of WGCNA co-expression module–trait associations. Values in each cell represent the correlation coefficient (r), with p-values in parentheses. Red and blue indicate positive and negative correlations, respectively; Fig. 10C: RDA biplot of metabolites, quality traits, and varieties. Arrows indicate the loading directions of quality traits, and different colored dots represent different varieties.

To reveal the associations between metabolites and quality traits at the “group” level, WGCNA was applied to cluster the 2038 putatively annotated metabolites into 14 co-expression modules, and the correlation coefficients between each module eigengene and the 9 quality traits were calculated (Fig. 10B). The MEgreen module showed the highest correlation with titratable acidity, a strong positive correlation with total phenolics, but negative trends with anthocyanin content, average fruit weight, appearance score, and texture score. This module mainly comprised flavonol glycosides and phenolic acids, suggesting that MEgreen may primarily participate in the regulation of phenolic and organic acid biosynthesis. Its negative correlation with anthocyanin content indicates that the anthocyanin biosynthetic pathway and the total phenolic biosynthetic pathway followed divergent dynamic trends. The MEtan module exhibited a strong positive correlation with anthocyanin content and a positive correlation with average fruit weight, but a negative correlation with total phenolics. The metabolic features of this module were coordinated with anthocyanin accumulation and berry size, suggesting its involvement in a specific regulatory network associated with fruit quality improvement. The separation from the total phenolic pathway further supports the specific regulation of the anthocyanin branch. The MEyellow and MEgrey modules showed moderate positive correlations with appearance scores, possibly associated with skin color and bloom. The MEblue module was negatively correlated with titratable acidity, whereas the MEturquoise module showed a positive correlation, suggesting that different metabolite modules may participate in the regulation of fruit acidity through distinct pathways.

To decipher the multivariate association patterns among metabolites, quality traits, and varieties at the “global structure” level, RDA was performed using the differential metabolites (VIP > 1 and |log₂FC| ≥ 1 from OPLS-DA) as explanatory variables (X) and the 9 major quality traits as response variables (Y) (Fig. 10C). The first and second RDA axes accounted for 58.02% and 16.37% of the raw constrained covariance, respectively, with a cumulative raw explained covariance of 74.39%. The overall adjusted constrained variance (adjusted-R^2^) after correction for the number of explanatory variables was 28.29%. Permutation tests indicated that both RDA1 and RDA2 were statistically significant. In terms of trait loadings, appearance score was located in the negative direction of RDA1, while titratable acidity, total phenolics, soluble solids, texture score, average fruit weight, flavor score, and anthocyanin were all positioned in the positive direction of RDA1, revealing a clear opposing differentiation between appearance quality and internal quality traits at the metabolic level. Anthocyanins and total phenolics showed consistent loading directions in the RDA space, corroborating the positive correlation between DENTICULAFLAVONOL and both traits observed in the correlation analysis, and further validating the network-level synergy between anthocyanin and phenolic metabolism.

The biological significance of key differential metabolites in grape berries is shown in Table 4. L-Phenylalanine, a key precursor of the phenylpropanoid pathway, was elevated in BG and positively correlated with anthocyanin accumulation. L-Methionine was up-regulated in all three hybrids, suggesting a shared metabolic basis for aroma precursor supply. In CG, the accumulation of L-malic acid and D-gluconic acid was associated with its superior sugar–acid balance. Peonidin-3-O-galactoside, a typical grape anthocyanin, was up-regulated in MG and consistent with its purple-red skin phenotype. Membrane lipid-related metabolites such as LysoPE and Glucosyl-C18-sphingosine were positively correlated with texture scores, providing a molecular basis for the crisp flesh trait of CG. These cross-validated results from multiple analytical dimensions improve the reliability of metabolite-trait associations.

**Table 4:** Key differential metabolites and their biological significance in grape berries.

Metabolite	RT (min)	Score	Level	VIP	*p*-value	FDR	Biological significance	Related quality traits
Kankanoside A	5.804	0.86	2	1.898	8.45 × 10^−15^	1.43 × 10^−12^	An iridoid derivative, a characteristic secondary metabolite of male parent ZHB, which is significantly over-expressed in MG	Rose aroma, flavor
10-Hydroxydecanoate	9.141	0.78	2	1.889	4.98 × 10^−6^	1.11 × 10^−5^	A medium-chain hydroxy fatty acid highly accumulated in BG and involved in lipid metabolism	BG-specific character, flavor
BRUCEOLIDE	5.679	0.53	2	1.465	9.57 × 10^−16^	3.90 × 10^−13^	A quassinoid alkaloid identified by the OPLS-DA model, participating in plant defense metabolism and contributing largely to inter-varietal metabolic divergence	Secondary metabolism, variety discrimination
Octyl 2-furoate	6.866	–	3	1.371	5.93 × 10^−6^	1.31 × 10^−5^	Furan ester with fruity/caramel-like aroma, positively correlated with flavor scores; it may contribute to the strong rose aroma and favorable flavor of MG	Flavor, rose aroma
Glucosyl-C18-sphingosine	9.997	–	3	1.338	1.16 × 10^−5^	2.34 × 10^−5^	A sphingolipid involved in cell-membrane micro-structure and signal transduction, and cooperatively regulates fruit firmness and softening together with cell-wall metabolism	Texture, flesh firmness
LysoPE(0:0/18:2(9Z,12Z))	8.796	0.76	2	1.203	2.30 × 10^−4^	3.47 × 10^−4^	A critical cell-membrane component for membrane assembly and stability. It is positively correlated with texture scores and provides a molecular basis for the crisp-flesh trait of CG	Texture, flesh firmness, crispness
Threonic acid	1.421	0.95	1	1.134	1.81 × 10^−4^	2.79 × 10^−4^	An ascorbate metabolite positively correlated with titratable acidity, participating in the co-regulation of fruit redox status and acidity	Titratable acidity, acidity
12-hydroxyjasmonic acid	5.734	0.68	2	1.047	4.51 × 10^−7^	1.39 × 10^−6^	A jasmonate derivative significantly positively correlated with titratable acidity. It may regulate organic-acid accumulation via hormone-signal pathways	Titratable acidity, acidity regulation

## 4 Discussion

This study compared three new table-grape cultivars with their parents at the DNA level, as well as in terms of fruit quality traits and metabolomic profiles. Newly-introduced cultivars undergo environmental adaptation, exhibiting significant differences in the bioactive components of food-medicine homologous substances(Suo et al., 2026).These findings provide important clues for understanding the molecular mechanisms of quality formation in hybrid offspring. The inheritance of fruit quality traits is generally complex, involving polygenic regulation and environmental influences (Dondini & Tartarini, 2014; Liu et al., 2024). The SSR marker analysis performed in this study not only confirmed the true hybrid nature of the three offspring varieties but also provided independent DNA-level evidence for the metabolomic clustering patterns observed. In this study, the soluble solids content of CG was similar to that of the female parent KG and significantly higher than that of the male parent ZHB, while the titratable acidity was significantly lower than both parents, demonstrating excellent sugar-acid balance with high sugar and low acidity. The morphological and biochemical characteristics of several grape hybrid combinations were evaluated at IARI, New Delhi. Among them, the hybrid ‘Pearl of Csaba’ × ‘Beauty Seedless’ exhibited the highest soluble solids content, the highest TSS/acid ratio, and the lowest titratable acidity, representing a typical excellent combination of high sugar and low acid (Sahoo et al., 2018). This type of heterosis for sugar-acid balance has important economic value in grape cross-breeding. Phenolics and anthocyanins are key functional components determining grape color, antioxidant capacity, and flavor complexity (Huang et al., 2025). Our study found that KG had the highest total phenolic content, BG showed significantly higher anthocyanin content than both parents, exhibiting obvious transgressive accumulation.The high contents of phenolics and anthocyanins in cultivars such as BG suggest that their processing by-products (e.g., peel and seeds) possess health-promoting application potential similar to grape seeds (Jebur et al., 2026; Seddiek et al., 2025). CG had relatively high anthocyanin content but low total phenolic level, indicating pathway divergence in phenolic synthesis in hybrid offspring. Similar to the phenolic-rich seed coat of walnut(Zhao et al., 2024), differences in phenolic composition among the grape cultivars in this study may likewise affect their sensory qualities (e.g., astringency) and nutritional functions. Significant differences in total phenolic content exist among grape varieties, and red interspecific hybrids can have even higher total phenolic content (Samoticha et al., 2017). Moreover, the metabolomic results further showed that flavonoids and anthocyanins were the core differential metabolites among varieties, highly consistent with the phenotypic measurements, confirming that activation of the anthocyanin biosynthesis pathway is the main reason for the deepening of skin color in purple-black varieties. In natural hybrid populations of European poplar (Populus), 41 QTLs for chlorogenic acid, salicin, and flavonoids were detected, demonstrating that the three branches of the phenylpropanoid pathway (chlorogenic acids, salicinoids, and flavonoids) have different heritabilities and genetic architectures, and are differentially affected by hybridization and evolutionary constraints; hybridization can lead to divergent responses of different branches of the phenylpropanoid pathway (Caseys et al., 2015; Chen et al., 2014). Total phenolic content was significantly positively correlated with sensory flavor scores, suggesting that phenolics not only enhance nutritional quality but also participate in flavor formation (Rodríguez-Lorenzo et al., 2023; Stoyanov et al., 2025). Sensory evaluation revealed that MG exhibited a rose-like aroma highly similar to its male parent ZHB. When both parents possess aroma characteristics, the heritability of aroma intensity in offspring is high, and the rose scent typically exhibits dominant inheritance (Tan et al., 2017). Using the rose-scented grape line ‘SP10140’ and the strawberry-scented line ‘SP122’ as parents, genetic analysis of volatile aroma components in 56 F1 hybrids revealed that the rose-scented parent contained abundant terpenoids (e.g., linalool, geraniol), and the composition and content of these aroma compounds underwent complex segregation and recombination in the hybrids (Suleiman et al., 2020), which is consistent with our finding that BG possesses both light rose and light strawberry aromas. MG had the highest comprehensive sensory score, with firm and thick flesh, good juiciness, and a rose aroma. Regarding texture, CG scored high for crispness, meeting the market demand for crisp-fleshed varieties, which is directly related to differences in organic acids and cell wall-related metabolites in its metabolome. This indicates that flavor traits of hybrid offspring can also be significantly improved through complementary desirable genes from both parents (Crespo et al., 2024). Appearance, flavor, and texture jointly determine commercial value; MG performed best due to its outstanding aroma, balanced sugar-acid ratio, and crisp flesh, indicating that aroma and flavor are the core drivers of improved sensory quality. The BG variety, with high anthocyanin content, represents a promising raw material for natural colorants and can serve as a plant-based alternative to synthetic food dyes in fruit juices, jams and fermented beverages. The KG variety has a total phenolic content of 14.26 mg/100 g, and phenolic-rich grape extracts possess multiple health-promoting benefits(Bhutani et al., 2025).

These five cultivars exhibited distinctly different metabolic profiles, and the random forest model could accurately discriminate among them, indicating that each cultivar possesses a unique metabolic fingerprint (Saha et al., 2026). Topi et al. used phenolic metabolite profiles of wines combined with a support vector machine to achieve 100% accurate classification of multiple grape varieties, noting that phenolics can serve as “robust chemical signatures.” The validity of metabolomic data as variety-specific metabolic fingerprints is supported by the fact that differences in flavonoids, amino acids, and organic acids among different varieties (including hybrids) constitute a stable and reliable basis for classification (Olivaro et al., 2025). PCA showed that PC1 and PC2 explained 54.2% of the total variance, with clear inter-group separation among varieties in the score plot, indicating that inter-variety metabolic differences were the main source of variation. Chatterjee et al. (Chatterjee et al., 2024) also achieved effective variety discrimination by PCA in a comparison of the Indian table grape hybrid MM with its parents, suggesting that PCA can serve as a rapid visualization tool for assessing genetic bias in the metabolome of hybrid offspring. Based on the OPLS-DA model, with VIP ≥ 1 and *p* < 0.05 as screening criteria, flavonoids and anthocyanins accounted for the highest proportion of differential metabolites, consistent with the differences in anthocyanin content measured in phenotypes. Using volcano plots to compare the asymmetric differential expression patterns between hybrid offspring and parents revealed that the inheritance of metabolic traits from parents by hybrid offspring is not simple additive inheritance (Bajpai et al., 2019; Zhang, Zhao, & Li, 2025). The cluster heatmap showed that MG clustered with the male parent ZHB, showing greater metabolomic similarity to the male parent; MG significantly overexpressed paternal characteristic terpenoid derivatives, which are key components of rose aroma. Koyama et al., using an interspecific hybrid population, found that the male parent ‘Muscat of Alexandria’ contained high concentrations of monoterpenes and norisoprenoids, and QTL analysis detected a major QTL controlling the concentration of 14 monoterpenes on linkage group 5 of the paternal genetic map (Koyama et al., 2022), providing direct genetic evidence for greater metabolomic similarity to the male parent of terpenoid metabolites. The contribution of different metabolite classes to the progeny does not follow a uniform pattern (Dan et al., 2025). CG clustered with the female parent KG, showing greater metabolomic similarity to the female parent and inheriting more of the flavonoid metabolic characteristics of KG. BG exhibited a “biparental recombinant” metabolic signature, without obvious bias toward either parent. These results confirm at the metabolome level that hybrid offspring are not simple additive combinations of parental traits but rather form specific traits through metabolic network remodeling, which is an important molecular basis for heterosis. The contribution of parents to the offspring metabolome may vary depending on the metabolite type (Bineau et al., 2022). In addition, epigenetic modifications in hybrid offspring may lead to imbalanced expression of parental alleles, thereby generating metabolic bias (Chen, 2013). BG did not clearly cluster with either parent, forming a unique metabolic profile, reflecting complex interactions between the parental metabolic networks and the activation of new metabolic pathways.

KEGG pathway enrichment analysis showed that for all three hybrid varieties and their parents, the differential metabolites were significantly enriched in the “Biosynthesis of amino acids” pathway, followed by secondary metabolic pathways such as “Flavone and flavonol biosynthesis”. Global remodeling of the amino acid metabolism network may be an upstream driving event for quality differentiation in hybrid offspring (Li, Yan, et al., 2022). Amino acids are not only substrates for protein synthesis but also precursors for many flavor substances (e.g., esters, terpenoids) and phenolic compounds (Zheng et al., 2022). In this study, the proportion of *L*-phenylalanine in BG was significantly higher than in both parents, and its anthocyanin content was also the highest, consistent with an enhancement of the phenylalanine metabolic pathway. The significant increase in L-malic acid and D-gluconic acid in CG is associated with its excellent sugar-acid balance; malic acid is one of the most important organic acids in grape berries, and its content directly affects fruit sourness and flavor balance (Michelini et al., 2021). Metabolites positively correlated with texture scores in this study, including LysoPE, glucosyl-C18-sphingosine, and 17a-Aza-D-homoandrost-5-en-3β-ol, are involved in cell membrane constituent assembly and cell wall metabolism, pathways governing fruit firmness and softening. These results are in agreement with findings from recent metabolomic studies, demonstrating that pectin composition and its enzymatic degradation constitute the primary determinants of texture differences in grape berries (Gan et al., 2026; Zhu, Wang, et al., 2026).

Differences in the flavonoid and anthocyanin synthesis pathways were key to distinguishing the metabolomes of the five varieties, and the specific upregulation of anthocyanins such as Peonidin-3-O-galactoside in MG was consistent with its purple-red skin phenotype. Figueiredo-González et al. found that peonidin-type anthocyanins are key discriminatory compounds distinguishing specific red grape varieties, and three red grape varieties could be distinguished with 100% accuracy based on their anthocyanin profiles. The inheritance tendency of peonidin-type anthocyanins in hybrid offspring is variety-specific and can be used to differentiate grape genotypes (Lu et al., 2021). The high phenolic content of KG may endow it with antioxidant functions comparable to those of *Phyllanthus emblica* polyphenols (Wu et al., 2025). Combined with the significant enrichment of the “flavonoid biosynthesis” pathway in the KEGG enrichment analysis, inter-cultivar differences in phenolic metabolism may constitute the metabolic basis for divergence in antioxidant capacity. Understanding metabolic trade-offs through integrative correlation analysis is critical for formulating breeding strategies that balance multiple traits. Modern consumer demands for table grapes increasingly take into account visual appearance, flavor quality and nutritional properties. The distinct metabolic architectures exhibited by BG, CG and MG demonstrate that cross-breeding can partially break negative correlations among various traits, making it feasible to develop new cultivars by pyramiding desirable traits from both parents.

## 5 Conclusion

This study systematically compared the differences in fruit quality and metabolome between table grape hybrid offspring and their parents. CG exhibited excellent sugar–acid balance, making it suitable for fresh consumption and juice processing. Based on their anthocyanin composition and sensory properties, BG and MG exhibit broad application prospects. SSR molecular markers confirmed that all three hybrids were true offspring, and each variety was assigned a unique SSR barcode-based molecular identity. PCA and OPLS-DA confirmed clear metabolomic separation among varieties, with flavonoids and anthocyanins identified as key differential metabolites. Correlation analysis revealed that flavonol glycosides showed strong positive correlations with anthocyanins and total phenolics; WGCNA analysis revealed that anthocyanins and total phenolics exhibited divergent dynamic trends; RDA analysis revealed an opposing differentiation pattern between appearance quality and internal quality traits at the metabolic level. KEGG enrichment analysis indicated that differential metabolites were mainly enriched in amino acid biosynthesis and flavonoid metabolism pathways, suggesting that amino acid metabolic remodeling is strongly associated with quality differentiation in hybrid offspring, thereby providing metabolic targets for improving grape fruit flavor, color, and nutritional quality through breeding. This study provides a theoretical basis for parental selection in table grape cross-breeding, while the SSR markers developed herein can be used for variety identification and intellectual property protection. The metabolomic data provide a reference for breeding grape varieties enriched in functional bioactive components such as phenolics and anthocyanins, with potential applications in the development of natural food ingredients and functional foods. Since the metabolite annotations are putative (MSI Level 2/3), targeted validation using authentic reference standards is still required. Future studies combining transcriptomics and allele-specific expression analysis are needed to further dissect the underlying genetic regulatory networks.

## CRediT authorship contribution statement

**Liu Zhenghai:** Writing – review & editing, Writing – original draft, Data curation. **Xiao Feng:** Visualization, Data curation. **Wang Chenyu:** Formal analysis, Data curation. **Qiao Qinghe:** Visualization, Data curation. **Liu Jinpin:** Investigation, Data curation. **Tan Min:** Writing – original draft, Data curation. **He Jinyu:** Formal analysis, Data curation. **Zhang Yanxia:** Investigation, Formal analysis. **Lu Jiang:** Writing – review & editing. **Ji Wei:** Writing – review & editing, Conceptualization. **Dong Zhigang:** Methodology, Funding acquisition. **Zhao Qifeng:** Supervision, Resources, Project administration.

## Informed consent statement

Informed consent was obtained from all subjects involved in the study.

## Institutional review board statement

This study does not collect sensitive personal data or involve commercial interests. All participants were adults, and their data were anonymized. Participants were fully informed and provided written consent prior to participation, with the option to withdraw at any time. In compliance with the “Ethics Review Measures for Life Sciences and Medical Research Involving Humans” issued by the National Health Commission of China in 2023 (Document No. 4), specifically Article 32, our study qualifies for exemption from ethical review as it does not present any potential risk to the participants.

## Ethics declaration

### Informed consent and patient details

Written informed consent to take part in the study and to publish the article has been obtained from all participants or their legal representatives. The privacy rights of participants have been observed.

### Studies in human

This study was performed in compliance with relevant laws, regulatory frameworks and guidelines where the research took place.

Ethics committee approval was not required under relevant laws and institutional guidelines. This study did not require ethical approval. All subjects participating in the sensory evaluation signed informed-consent and confidentiality agreements, consenting to the use of their data for research purposes. All personal information of participants was kept confidential to protect individual privacy and comply with legal standards.

## Funding

This research was funded by The Biological Breeding Project of Shanxi Agricultural University (YZGC2026054); The Key Research and Development Program of Ningxia Hui Autonomous Region (2024BBF01002); The Key Research and Development Project of Shanxi Province on Forestry and Grassland (JLYF-2025-31);Shanxi Provincial Basic Research Program Project (202303021212098); Open Project of the Key Laboratory of Modernization of Traditional Chinese Veterinary Medicine (Grant No. SXKL2025004); Ministry of Finance and Ministry of Agriculture and Rural Affairs: Agriculture Research System of China (CARS-29-yz-5).

## Declaration of competing interest

The authors declare that they have no known competing financial interests or personal relationships that could have appeared to influence the work reported in this paper.

## Data Availability

The original contributions presented in the study are included in the article, further inquiries can be directed to the corresponding author/s.
