# Supplementary material for: Comparison of fruit quality and metabolome differences between three table grape varieties and their parents
Source: Food Chem X. 2026 Oct 15;40:104567. doi: 10.1016/j.fochx.2026.104567 (PMC13694753; doi:10.1016/j.fochx.2026.104567)
Supplement: Supplementary file 1 [file mmc1.docx]

Table S1 Test Chemicals

| **Reagent name** | **Manufacturer** | **Purity** |
| --- | --- | --- |
| Methanol | Thermo Fisher | LC-MS grade, ≥99.9% |
| Formic acid | Thermo Fisher | LC-MS grade, ≥99.9% |
| Ammonium acetate | Thermo Fisher | LC-MS grade, ≥99.9% |
| Hi‑Di deionized formamide | ABI | Analytical reagent grade (AR), ≥99.5% |
| POP‑7 Polymer | ABI | AR ≥99.5% |
| GS‑500LIZ Size Standard | ABI | Highly deionized |
| 10×Buffer | ABI | Sequencing grade |
| 2× Taq plus PCR MasterMix | Ruibio | Molecular biology grade |
| PCR plate | AXYGEN | Molecular biology grade |
| Genomic DNA Extraction Kit | Ruibio Biotechnology (Beijing) | Molecular biology grade |
| Sodium hydroxide | Sinopharm Chemical Reagent Co., Ltd. | AR ≥99.5% |
| Sodium carbonate | Sinopharm Chemical Reagent Co., Ltd. | AR ≥99.5% |
| Sodium acetate | Sinopharm Chemical Reagent Co., Ltd. | AR ≥99.5% |
| Potassium chloride | Sinopharm Chemical Reagent Co., Ltd. | AR ≥99.5% |
| Ethanol | Sinopharm Chemical Reagent Co., Ltd. | AR ≥99.5% |
| Catechin standard | Sinopharm Chemical Reagent Co., Ltd. | LC-MS grade, ≥99.9% |
| Experimental water | Wahaha Group Co., Ltd. | Ultrapure water |

Table S2 Test Equipment

| **Instrument name** | **Manufacturer** | **Model and specification** |
| --- | --- | --- |
| Centrifuge | Eppendorf | Centrifuge 5810R |
| Genetic Analyzer | ABI | 3730xl DNA analyzer |
| Capillary | ABI | 50 cm, 96‑capillary array, 4331246 |
| PCR instrument | ABI | 2720 |
| Electrophoresis apparatus | Beijing Liuyi Instrument Factory | DYY‑8C |
| UV analyzer | Beijing New Technology Application Research Institute | UV‑IV |
| Vortex mixer | Qilinbeier Instrument Manufacturing Co., Ltd., Haimen | VORTEX‑5 |
| Grinder | Beijing Hede Technology Co., Ltd. | Jing N.9548 |
| Dual‑beam ultraviolet spectrophotometer | Shanghai Aoxi Scientific Instrument Co., Ltd. | UV1902 |
| Digital display double‑row six‑hole water bath | Shanghai Wanning Company | HH‑6 |
| TPL‑1 Digital Refractometer | ATAGO | TPL‑1 |
| Mass spectrometer | Thermo Fisher | Q Exactive™ HF / Q Exactive™ HF‑X |
| Chromatograph | Thermo Fisher | Vanquish UHPLC |
| Chromatographic column | Thermo Fisher | Hypersil Gold column (100×2.1 mm, 1.9 μm) |
| Refrigerated centrifuge | Scilogex | D3024R |

Table S3 List and sequences of 30 pairs of SSR primers

| **Primer No.** | **Primer name** | **Allelic variant** | **Chromosome** | **Primer sequence (5′→3′)** |
| --- | --- | --- | --- | --- |
| P01 | VVMD28 | 215  225  255 | 3 | F:ACAATTCAATGAAAAGAGAGAGAGAGAR:TCATCCATTTTCGTATCTCTATTTGCTG |
| P02 | VVMD32 | 236  248  268 | 4 | F:GGAAAGATGGGATGACTCGCR:TATGATTTTTTTAGGGGGGTGAGG |
| P03 | VVMD27 | 177  185 | 5 | F:TACCAGATCTGAATACATCCGTAAGTR:ACGGGTATAGAGCAAACGGTGT |
| P04 | VrZAG79 | 240  242  244  256 | 5 | F:AGATTGTGGAGGAGGGAACAAACCGR:TGCCCCATTTTTCAAACTCCCTTCC |
| P05 | VVMD7 | 237  241  247 | 7 | F:AGAGTTGCGGGAGAACAGGATR:CGAACTTTCACACGCTTGAT |
| P06 | VrZAG62 | 184  186  194 | 7 | F:GGTGAAATATGGGCCGAACACACGCR:CCATGTCTCTCTCAGCTTCTCAGC |
| P07 | VVMD25 | 236  246  252 | 11 | F:TTCCGTTAAAGCAAAAAGAAAAAGGR:TTGGATTTGAAATTTATTGAGGGG |
| P08 | VVS2 | 133  135  141 | 11 | F:CAGCCCGTAAATGTATCCATCR:AAATTCAAAATTCTAATTCAACTGG |
| P09 | VVMD5 | 122  124 | 16 | F:CTAGAGCTACGCCAATCCAAR:TATACCCAAAATCATATTCCTAAA |
| P10 | VMC4F8 | 231  233  235 | 1 | F:CATTCATAGGGTTTTTCACAGCR:CTGCCAGTATACTGATTCCTCTC |
| P11 | VrZAG93 | 116  118  122  124 | 2 | F:GCACTTCTTGACGTTTAAAACAGCCR:TATGGAGGGACCGAGGTGGGCTAGG |
| P12 | Vchr3a | 184  210 | 3 | F:CAATCATATGAGCAAGGCATGTR:GCTTCCTGAAAATTTGTGTGCCA |
| P13 | Vchr4a | 177  185  185 | 4 | F:CAACTGGGATCCAAGACCTCR:CAGCTTCACAGGTAAACCACA |
| P14 | Vchr6a | 170  178 | 6 | F:AATGTTGAGCTTTTGGGGCTTGR:CCAATTTCCCATACATCTCAAAA |
| P15 | VVMD21 | 241  245  263 | 6 | F:GGTTGTCTATGGAGTTGATGTTTGCR:GCTTCAGTAAAAAGGATTTGCG |
| P16 | VVIb66 | 84  92  94  98 | 8 | F:CCACTAGTGGTCAGAAAAAGAAGR:TTGTATTGTGTGCTCTCTTCTCA |
| P17 | Vchr8a | 171  193  205 | 8 | F:ACCACTGCCACTCTCATR:AAATCTCCGGGATCCTTTTG |
| P18 | Vchr9b | 114  132 | 9 | F:AGCGTCATGACAGGGATTCAGAAR:AAAGAATTAATCATTAACCATTCACG |
| P19 | VMC1C10 | 145  157  183 | 9 | F:CACAGCTGTTCCAAGTCCCAR:ACAAGCTTCCGGCACTCTC |
| P20 | VrZAG67 | 136  148  150 | 10 | F:ACCTGGCCGACTCCTCTTGTATGCR:TCCTGCCGCGGATAACCAAGCTATG |
| P21 | VMC4F3‑1 | 168  174  186  198 | 12 | F:AAAGCACTATGGTGTGGGTGTAAAR:TAACCAATACATGCATCAAGGA |
| P22 | Vchr13b | 140  152 | 13 | F:TAAGCATTCTGGGCGCTTTTCCR:TCGTCTATATGCGACCTTGG |
| P23 | Vchr13c | 114  120  123 | 13 | F:AGACCCAAGGGGCAAGGTACTR:AACACCGTTAGGGCATACTCCA |
| P24 | VMC5G8 | 330  311  313 | 15 | F:CATGCACATCTTGTTTCACTCTR:CATCATTGCTTCCAAAAAGTCTC |
| P25 | Vchr16a | 107  119  161  169 | 16 | F:TTCATGTGTGACACCCCTTTR:AATGTCCATGCTTCAAAAATACC |
| P26 | Vchr17a | 183 | 17 | F:AGGAAGAGGATTGATCACCAR:GTGCCAACGCTTGCACTATT |
| P27 | SCU06 | 167  171 | 17 | F:CCTAATGCCAGGAAGGTTGCR:CCCTAGTCTCTCTACCTATCCATG |
| P28 | Vchr18a | 149  161 | 18 | F:TTCCCCACCCCGGTAAATATGAR:CATCCAAAACATCACGCTGAG |
| P29 | VVIP31 | 171  177  181  183 | 19 | F:TATCCCAAGAGACAAATTTCCCACR:TTCTCTTGTTTCCTGCAAAATGG |
| P30 | Vchr19b | 160  164 | 19 | F:TTTGTTAGGTGTTTGTTACCCGGTAR:ATCTTCTGGCCATGTGGTTTC |

## 4. Permutation test report for OPLS‑DA

The dataset was divided into a training set, validation set and test set. The training set was used for model construction, the validation set for model optimization, and the test set to assess the predictive performance of the model. Seven‑fold cross‑validation was adopted. Briefly, the dataset was split into 7 subsets. One subset was selected as the validation set each time to evaluate model prediction performance, while the remaining six subsets served as the training set for model building. This procedure was repeated until all samples had been predicted. The predicted group labels were compared with the true group labels to calculate the predicted residual sum of squares (PRESS). For convenience, PRESS was converted into Q2 (1 − PRESS/SS). The values of Q2 and R2Y for the final model correspond to the y‑axis values at x = 1 in Figure S1. Overfitted models usually yield high R2Y (representing the model’s fitting performance on the training set), whereas Q2 remains low. For biological samples, Q2 ≥ 0.4 is considered satisfactory; Q2 ≥ 0.2 is often acceptable, though the model has weak predictive power. During autofit in the ropls package , the number of predictive components is determined based on Q2 (for OPLS‑DA models, the number of orthogonal components). Components are added incrementally, and the algorithm stops adding components when Q2 plateaus or decreases.

Group labels of samples were randomly permuted, and models were reconstructed to compute R2Y and Q2Y. This permutation was repeated 200 times. If the resulting pR2Y and pQ2 are less than 0.05, the model can be regarded as reliable.

Figure S1 Permutation test plot of the OPLS‑DA model
